# Development and application of an in-house library and workflow for gas chromatography–electron ionization–accurate-mass/high-resolution mass spectrometry screening of environmental samples

**DOI:** 10.1007/s00216-021-03810-w

**Published:** 2021-12-04

**Authors:** Verónica Castro, José Benito Quintana, Javier López-Vázquez, Nieves Carro, Julio Cobas, Denis Bilbao, Rafael Cela, Rosario Rodil

**Affiliations:** 1 Department of Analytical Chemistry, Institute of Research On Chemical and Biological Analysis (IAQBUS), Universidade de Santiago de Compostela, 15782 Santiago de Compostela, Spain; 2 INTECMAR - Technological Institute for the Monitoring of the Marine Environment of Galicia, Peirao de Vilaxoán S/N, 36611 Vilagarcía de Arousa, Spain; 3 Department of Analytical Chemistry, University of the Basque Country (UPV/EHU), 48940 Leioa, Spain; 4 Research Centre for Experimental Marine Biology and Biotechnology, University of the Basque Country (PiE-UPV/EHU), 48620 Plentzia, Spain

**Keywords:** Gas chromatography–quadrupole time of flight (GC-QTOF), Contaminants of emerging concern (CECs), Persistent organic pollutants (POPs), Kovats retention index, Matrix solid-phase dispersion (MSPD)

## Abstract

This work presents an optimized gas chromatography–electron ionization–high-resolution mass spectrometry (GC-EI-HRMS) screening method. Different method parameters affecting data processing with the Agilent Unknowns Analysis SureMass deconvolution software were optimized in order to achieve the best compromise between false positives and false negatives. To this end, an accurate-mass library of 26 model compounds was created. Then, five replicates of mussel extracts were spiked with a mixture of these 26 compounds at two concentration levels (10 and 100 ng/g dry weight in mussel, 50 and 500 ng/mL in extract) and injected in the GC-EI-HRMS system. The results of these experiments showed that accurate mass tolerance and pure weight factor (combination of reverse-forward library search) are the most critical factors. The validation of the developed method afforded screening detection limits in the 2.5–5 ng range for passive sampler extracts and 1–2 ng/g for mussel sample extracts, and limits of quantification in the 0.6–3.2 ng and 0.1–1.8 ng/g range, for the same type of samples, respectively, for 17 model analytes. Once the method was optimized, an accurate-mass HRMS library, containing retention indexes, with ca. 355 spectra of derivatized and non-derivatized compounds was generated. This library (freely available at https://doi.org/10.5281/zenodo.5647960), together with a modified Agilent Pesticides Library of over 800 compounds, was applied to the screening of passive samplers, both of polydimethylsiloxane and polar chemical integrative samplers (POCIS), and mussel samples collected in Galicia (NW Spain), where a total of 75 chemicals could be identified.

**Supplementary Information:**

The online version contains supplementary material available at 10.1007/s00216-021-03810-w.

## Introduction

Sustainable development is seriously impaired by the amount of many different chemicals which are emitted into our environment. Thus, chemical pollution has been considered one of the planetary boundaries, and closely linked to another one (freshwater, since the availability of safe freshwater resources is seriously threatened by chemical pollution), where action is needed [1–4]. In this context, we need efficient analytical methods, such as high-resolution mass spectrometry (HRMS)–based screening methods, capable of providing information on a broad range of chemical pollutants [5, 6]. Such screening methods can be used, for instance, as a first step in prioritizing the compounds that could later be determined at a quantitative level and in order to provide a broader picture of the chemical pollution space. In this context, liquid chromatography–HRMS (LC-HRMS) has become the most prominent technique in environmental screening analysis [7–14]. However, LC-HRMS has a limited applicability for several compounds which exhibit poor ionization efficiency by electrospray. Atmospheric pressure chemical ionization (APCI) and atmospheric pressure photoionization interfaces have been shown to be able to expand the capabilities of LC-(HR)MS to more hydrophobic compounds [15–17], but, so far, have found limited use in HRMS screening studies. Hence, gas chromatography–HRMS (GC-HRMS) is a good complement to LC-HRMS, having as further advantages the fact of being an ideal technique for volatile analytes, its higher separation power, and being less prone to matrix effects. An additional advantage of GC is that linear retention indexes (RI) are already available for several chemicals, further providing confirmatory confidence of compounds identity [18].

Although some researchers proposed a screening workflow in GC-HRMS systems equipped with an APCI source very similar to LC-HRMS, this is typically performed by deconvolution of chromatograms obtained by (both low resolution and HRMS) GC–MS instruments equipped with classical electron impact (EI) sources. Deconvoluted spectra can be compared to libraries with a high probability to obtain a good match as compared with non-deconvoluted spectra. The NIST MS library is by far the most frequently employed one in GC–MS screening studies [19–23]. However, it has the inconvenience of containing low-resolution mass spectra. Moreover, conversely to LC–MS, the availability of accurate-mass HRMS libraries for GC-EI-MS is very limited.

Deconvolution of GC-HRMS data processing has evolved significatively due to the introduction of new powerful, both commercial (e.g., Agilent MassHunter Unknowns Analysis) and open-source (e.g., MZmine2), software. Several papers have been published in order to improve the data mining workflow using open-source software such as MZmine2 [24–26]. However, poor information is available dealing with commercial software workflows, such as Agilent MassHunter Unknowns Analysis. For a specific chromatogram, the deconvolution algorithm (of this last software) can generate hundreds or even thousands of detected peaks. Therefore, it is important to appropriately select the deconvolution parameters in order to minimize the number of false positives and negatives. In the literature, there are a limited number of screening studies published that describe the use of the Unknowns Analysis software for data treatment, e.g., [20, 27, 28], and only one of them optimized a single parameter (the narrow extracted mass window (EMW)) [27] affecting the identification process of the deconvolution algorithm. EMW is not currently used in the most recent versions of this software, as the deconvolution algorithm was completely redesigned to work with HRMS data acquired in profile mode.

The main goals of this study were the optimization of a GC-HRMS deconvolution workflow algorithm (Agilent Unknowns Analysis) and its application to coastal mussel samples and coastal/marine- and freshwater-deployed passive samplers. To this end, first of all, an EI-HRMS library of 26 model compounds (Table S1) was created and employed in order to find out the best operating conditions in terms of false positive/false negative balance with spiked marine biota samples (mussel) extracts. Once the method was optimized, an accurate-mass HRMS library, totalling 356 different spectra, including derivatized and non-derivatized compounds, was generated and employed, together with an Agilent EI-HRMS (modified) library of pesticides to a set of environmental samples. Such samples included passive samplers and mussels, because of their ability to capture chemical pollutants along time. Passive samplers, including, among others, polar organic chemical integrative samplers (POCIS) and polymeric materials like low-density polyethylene (LDPE) and polydimethylsiloxane (PDMS) [13, 29–32], have been successfully used for water monitoring, including screening studies. Bivalve molluscs, such as mussels, are an important filter feeding organisms which have been used as bioindicators in environmental monitoring programs [33–35] due to their capacity to accumulate contaminants.

## Materials and methods

### Reagents and sorbents

Dichloromethane (DCM) for pesticide residue analysis was provided by VWR Chemicals (Radnor, PA, USA). Acetonitrile (ACN) hypergrade for LC–MS, ethyl acetate (EtOAc) for GC–MS, and isooctane for GC were provided by Merck (Darmstadt, Germany). Sulfuric acid (H_2_SO_4_) 95–98% was provided by Panreac (Barcelona, Spain). The silylation reagent, N-methyl-N-(trimethylsilyl) trifluoroacetamide (MSTFA), and the C_7_–C_40_ saturated alkanes standard solution were provided by Sigma-Aldrich (San Louis, MO, USA). Methanol optima LC–MS grade was provided by Thermo Fisher Scientific (Waltham, MA, USA). Ultrapure water was obtained in the laboratory by purifying demineralized water in a Milli-Q Gradient A-10 system (Merck-Millipore, Bedford, MA, USA).

Florisil (60–100 mesh) and primary-secondary amine–bonded silica (PSA) were provided by Supelco (Bellefonte, PA, USA). Silica gel 60 (0.040–0.063 mm) was provided by Merck. Oasis HLB 12 cc (500 mg) cartridges were supplied by Waters (Milford, MA, USA). Polyethersulfone (PES) membranes Suport®-450 47 mm 0.45 µm were obtained by Pall Corporation (Ann Arbor, MI, USA). Polydimethylsiloxane (PDMS) elastomer with 2 mm diameter was from Goodfellow (Huntingdon, UK). Florisil and silica were washed in a PLE system using an ASE 200 (Dionex, Idstein, Germany) apparatus, equipped with 33 mL stainless steel extraction cells, using first acetonitrile and then ethyl acetate at 60 °C, and then dried into the oven at 120 °C for 24 h, in order to minimize blank contamination issues [36].

POCIS stainless steel holders were constructed by Nodosafer (Pontevedra, Spain) with 70 mm external diameter and 40 mm internal diameter. Before POCIS assembly, stainless steel material was washed with soap, Milli-Q water, and methanol. PES membranes were sonicated in methanol and PDMS elastomer in ethyl acetate three times each one (10 min each time) in an ultrasonic bath and dried at room temperature. The HLB sorbent was obtained from Oasis HLB 12 cc cartridges. Oasis HLB cartridges were conditioned with 20 mL of methanol and dried under nitrogen stream before being dismantled. POCIS was assembled enclosing 100 mg of HLB sorbent between two PES membranes. The sandwich was sealed with two stainless steel rings and bolted with three stainless steel screws [13].

Pieces of 50 cm of PDMS were cut and rolled around one stainless steel ring. POCIS devices and PDMS were placed in stainless steel cages with holes that let water run through and protect them against potential damages on the sampling points.

### Sampling and deployment of passive samplers

Mussel (*Mytilus galloprovincialis*) samples were collected on the Atlantic estuaries Ría de Arousa (coded A1-A3) and Ría de Vigo (coded V1-V3) located in the northern coast of Spain in 2019 (Figure S1). Mussels were homogenized and freeze-dried in amber glass bottles by the Galician Technological Institute for the Monitoring of the Marine Environment (INTECMAR) and sent to the University of Santiago de Compostela for analysis.

Steel canisters with the passive samplers were placed in 3 locations in rivers (coded R1-R3) and 4 locations in the estuary Ría de Arousa (coded S1-S4) from Galicia (northwest of Spain) during 2019 (Figure S1) for 1 and 2 weeks, respectively.

### Sample treatment

#### Passive samplers’ desorption

At the end of the sampling period, passive samplers were collected and transported to the lab. After reception, POCIS and PDMS were cleaned with abundant Milli-Q water, wrapped in aluminum foil, and stored at − 20 °C until desorption.

POCIS were disassembled and particles of HLB sorbent were transferred into an empty SPE cartridge and packed between two frits. Compounds adsorbed in the POCIS sorbent were eluted by gravity using 10 mL of methanol [13].

PDMS desorption was carried out with 15 mL of ethyl acetate in a vial by shaking at 175 rpm for 60 min using an orbital shaker supplied by Science Basic Solutions (SBS) (Rubí, Spain) [37].

POCIS and PDMS extracts were concentrated to ca. 0.5 mL in a Turbovap II concentrator (Zymark, Hopkinton, MA), then evaporated to dryness under a purified nitrogen stream and finally reconstituted in 500 µL and 1000 µL of ethyl acetate, respectively. An aliquot of 75 µL of each extract was derivatized with 25% of MSTFA by heating in the oven for 60 min at 65 °C [38].

With each set of samples, procedural blanks of POCIS and PDMS were performed and submitted to the corresponding protocols.

#### Mussel sample extraction

Freeze-dried mussel samples were processed using two different matrix solid-phase dispersion (MSPD) methods, as to cover analytes with different properties. In the first one (method A), based on [39], 0.5 g of freeze-dried mussel sample was mixed into a glass mortar with 0.5 g of PSA. The homogenized mixture was transferred into a cartridge containing different cleanup sorbents (0.5 g of silica followed by 1.75 g of acidified silica (10% (w/w) H_2_SO_4_) and 1.75 g of Florisil (deactivated with 5% (w/w) H_2_0). Analytes were eluted with 10 mL of dichloromethane. The eluate was concentrated to dryness under a nitrogen stream and reconstituted in 200 µL of isooctane.

In the second method (method B), based on [36], 0.5 g freeze-dried mussel was mixed with 1.2 g activated silica into a glass mortar. The homogeneous mixture was transferred into a cartridge containing 3 g of deactivated (5% (w/w) H_2_O) Florisil. Analytes were eluted with 10 mL of acetonitrile. The extract was concentrated into a Turbovap II concentrator and evaporated to dryness under a purified nitrogen stream and reconstituted in 100 µL of ethyl acetate. An aliquot of 50 µL of each extract obtained by method B was derivatized with 25% of the silylating reagent MSTFA by heating in an oven for 60 min at 65 °C [38].

Procedural blanks of both MSPD methods were performed with each batch of samples.

### Gas chromatography–high-resolution mass spectrometry

A GC-HRMS system comprised of a 7890A gas chromatograph, a 7638B automatic sampler, and a 7200 quadrupole time-of-flight (QTOF) mass spectrometer from Agilent Technologies (Wilmington, DE, USA) was employed. The system was controlled by MassHunter Acquisition B.07.06 software (Agilent).

Chromatographic separation was carried out on an HP-5MS capillary column (30 m × 250 µm i.d., 0.25 µm film thickness) supplied by Agilent Technologies. High-purity helium (99.9999%, Nippon Gases, Madrid, Spain) was used as carrier gas at a constant flow rate of 1 mL/min. The temperatures of the transfer line, quadrupole, and source were set at 280 °C, 150 °C, and 230 °C, respectively. Oven temperature was programmed as follows: 50 °C (held for 1 min) ramped at 10 °C/min to 290 °C (held for 15 min). The total run time was 40 min and the solvent delay 3.5 min. Injections of 1 µL were made at 280 °C in splitless mode for 1 min using a 10 µL syringe. The injector was equipped with an Agilent ultra-inert liner containing glass wool.

The QTOF mass spectrometer was operated at 2-GHz in the EI mode at 70 eV with the emission current filament set at 5 μA and in single MS mode. Data was acquired in both centroid and profile mode in the range from 40 to 1000 m*/z*, at a frequency of 5 spectra/s [27] and providing a full width at half maximum (FWHM) resolution of ca. 5700 at 68 m*/z* and ca. 8400 at 502 m*/z*. The QTOF mass axis was automatically recalibrated every 3 injections by infusion of a commercial solution of perfluorotributylamine in the EI source.

### Data analysis and in-house libraries for suspect screening

Data analysis was carried out with the Agilent MassHunter Unknowns Analysis B.10.00 software using the SureMass algorithm. Features were extracted by spectral deconvolution and the spectra for each feature were compared with those of a HRMS spectral library. In addition, retention indexes (RI) were also employed. Deconvolution, identification, and library search optimal parameters are described in Table 1.

**Table 1:** Parameters of the Unknowns Analysis method employed for the screening of organic pollutants driven by accurate-mass libraries

Peak detection and deconvolution	
Algorithm	SureMass
Absolute area ( ≥)	10,000 counts
RT window size factor	25, 50, 100, 200
Extraction window, *m/z* delta	0.05 AMU
Min. number of ion peaks	3
Max. number of ion peak shapes to store	10
Integrator	Agile 2
**Library search**	
Libraries	In-house library & Agilent RTL Pesticides Library
Pure weight factor	0.1
RT penalty function	Trapezoidal
RT range (s)	20^a^/30^b^
Penalty-free RT range (s)	20^a^/30^b^
RT mismatch penalty	Multiplicative
Max RT penalty	20^a^/30^b^
Accurate mass tolerance (ppm)	50
**Compound identification**	
Max hit count	1
Min match factor	75
Min m/z	30
Library search type	Spectral search

^a^Parameter used with in-house library; ^b^Parameter used with the modified pesticide library

For the analysis of environmental samples, two HRMS libraries were used: a commercial Agilent RTL Pesticides Library (modified to include RIs, see below) and the in-house library. A total of 300 compounds were selected for the creation of the in-house library. Standard solutions of ca. 1000 ng/mL were injected along with a C_7_–C_40_ alkane mix standard under the same chromatographic conditions, to obtain the spectra of individual compounds and calculate the corresponding Kovats RI. For 239 compounds, acceptable peak shape, retention, and intensity were obtained. Detailed information on name, CAS number, RI, and class from these individual contaminants is reported in Table S2. Moreover, for those compounds with reactive functional groups, an aliquot of standard was derivatized with a 25% of MSTFA by heating in an oven for 60 min at 65 °C [38]. Table S3 contains the 116 derivatized compounds, among which a non-derivatized spectrum was also available for 55 chemicals, while 61 of them produced a chromatographic peak only when derivatized. These 61 compounds include 26 pharmaceuticals, 20 human metabolites, 8 pesticides, 4 additives, and 3 transformation products. This library covers organic chemicals with molecular weights ranging from 104 to 692 Da, log *K*_ow_ from − 1.2 to 9.6 and RIs from 900.5 to 3257.

The commercial library Agilent Pesticide AMRT PDCL (Table S4), which contains retention time (RT) values (when using a particular set of column and temperature program), was modified by including RIs. To this end, 12 chemicals with a wide range of retention times (RIs ranging from 1192 to 3257) and the n-alkanes mix were injected in the GC-QTOF with the Agilent reference oven program. Library retention time values were then updated using a linear correction between the library and the experimentally obtained retention times, and then converted into RIs.

A calibration retention time (CRT) file was generated for each set of samples using the C_7_–C_40_ n-alkane standard mix injected under the same chromatographic conditions. The CRT file is a csv file that contains the name, retention time, and the Kovats RI of each alkane and is used by the Unknowns Analysis software for RI matching.

### Optimization of Unknowns Analysis deconvolution parameters

Several parameters of the Unknown Analysis method using the SureMass algorithm were optimized in order to achieve the best compromise between false positives and false negatives. To this end, a set of 26 model different compounds (see Table S1) was employed for the creation of an EI-HRMS library by injection of individual standards. These chemicals belong to different families (fragrances, plasticizers, phthalates, polycyclic aromatic hydrocarbons (PAHs), parabens, polybrominated diphenyl ethers (PBDEs), benzothiazoles, pesticides, polychlorinated biphenyls (PCBs), organophosphate esters, and UV filter compounds), with molecular weights between 128 and 481 Da and log *K*_ow_ between 1.7 and 8.0. It also contains compounds with poor (in terms of number of ions) spectra (e.g., dibutyl phthalate, pyrene, and benzothiazole) and rich spectra (e.g., tris(2-butoxyethyl)phosphate, 4-methyl-1H-benzotriazole and triclosan), and a retention time range between 6.3 min (2-chlorophenol) and 26.3 min (benzo[a]pyrene), chemicals with and without Cl/Br atoms, and compounds showing a good peak shape (e.g., BHT) or tailing peaks (e.g., 2-chlorophenol).

Five replicates of mussel extracts (obtained by method B, as detailed above) were spiked with a mixture of these 26 compounds at two concentration levels (10 and 100 ng/g dry weight (dw) in mussel, equivalent to 50 ng/mL and 500 ng/mL in extract) and injected in the GC-QTOF system. Compound identification was performed by considering different method parameters (accurate mass tolerance (AMT), match factor, and pure weight factor (PWF)). From these parameters, AMT represents the maximum allowed difference between target and library exact masses; the score represents the cutoff value (100% is a perfect match) for positive identification, and PWF controls the forward and reverse matching balance, where the values of 0 and 1 represent a purely reverse and forward search, respectively, and the values in between a weighted combination of those extreme situations. AMT and match factor were first assessed at the highest concentration level in terms of false negatives, while the most critical parameter, PWF, was then optimized at the lowest concentration, where both false positives and negatives are considered. Retention times and RIs were not considered at this stage since we wanted to select the best conditions purely in spectral matching terms.

### Method performance and quantitative analysis

From the compounds found in both water and mussel samples, 17 chemicals, whose standards were available in the lab, were selected for method performance evaluation. Mussel samples with low contamination levels and passive sampler sorbents were spiked with the selected compounds at two concentration levels (5 and 50 ng/mL referred to the extract, equivalent to 1–2 and 10–20 ng/g dw, respectively, depending on the MSPD method), analyzed (five replicates) together with the respective non-spiked samples and blanks according to the described procedures, submitted to the GC-EI-HRMS optimized screening workflow, and, finally, the percentage of chemicals positively detected was calculated. Then, the screening detection limit (SDL) was established as the lowest concentration for which it has been demonstrated that an analyte can be detected in at least 95% of the samples [40].

Trueness and LOQ evaluation for these 17 chemicals was performed by spiking passive sampler extracts or mussel samples. Trueness (recovery and repeatability) was evaluated at one concentration level (50 ng/mL referred to standard, equivalent 10–20 ng/g dw when referring to mussel samples). An estimation of their concentration in the samples was then performed by the external standard calibration method (LOQ-100 ng/mL calibration range).

## Results and discussion

### Optimization of the deconvolution parameters

#### Accurate mass tolerance

As a preliminary step, we optimized the AMT, which represents the maximum mass error (in ppm) allowed by the software during comparison of deconvoluted and library spectra. Thus, the samples spiked at 500 ng/mL (100 ng/g dw referred to sample) were processed with the Unknowns Analysis SureMass deconvolution algorithm with three different AMT values (10 ppm (default value), 20 ppm, and 50 ppm) combined to five different values of PWF (0, 0.25, 0.5, 0.75, and 1) and setting the minimum match factor to 50 (this value, in percentage, refers to spectral match against the library). Figure S2 shows the percentage of false negatives (compounds not being detected) in each combination of AMT and PWF. As observed, no compounds were identified at all using the default value of 10 ppm at any PWF. By increasing the AMT value, the percentage of false negatives decreased, being in the range 43–50% at 20 ppm and 0% at 50 ppm for any PWF. Therefore, the AMT was fixed at this latest value of 50 ppm. This relatively large AMT value is mostly due to the fact that the software used for creation of the library and the SureMass algorithm used during deconvolution perform a different interpolation for the conversion of profile to centroid spectra. Besides, other factors related to this are the limited resolution of the GC-QTOF and the fact that EI spectra contain many low masses. It is expected that software upgrading and improved performance of the latest generation of GC-EI-QTOF systems will help into reducing the AMT in the near future.

#### Establishment of match factor cutoff values

Once the AMT was set, the minimum library match factor for the identification of the chemicals was evaluated at six different values of PWF (0, 0.1, 0.25, 0.5, 0.75, and 1) with the samples spiked at the highest concentration level (500 ng/mL referred to extract, 100 ng/g dw referred to sample). The distribution of match factor values obtained at the different PWFs is presented as Box-and-Whisker plots in Figure S3. Thus, the match factor cutoff values allowing a 0%, 1%, and 5% of false negatives could be established as the values presented in Table 2. As it can be observed, lower match factor cutoff values were required at higher PWFs (i.e., when forward search poses higher weights) for the positive identification of the analytes. The ideal experimental parameters should enable a 0% of false negatives but, unfortunately, this rarely happens. Hence, in agreement with the recommendation of the SANTE/12682/2019 guide [41] for screening methods, 5% false negatives cutoff values were used to further evaluate PWFs.

**Table 2:** Values of match factor allowing 0%, 1%, and 5% of false negatives (mussel spiked concentration: 100 ng/g dw, equivalent to 500 ng/mL in the extract) for different values of pure weight factor (PWF)

PWF	0% false negatives	1% false negatives	5% false negatives
0	73	74	76
0.1	73	73	75
0.25	72	72	73
0.5	57	61	70
0.75	43	53	67
1	29	54	64

#### Selection of pure weight factor

**Fig. 1:**
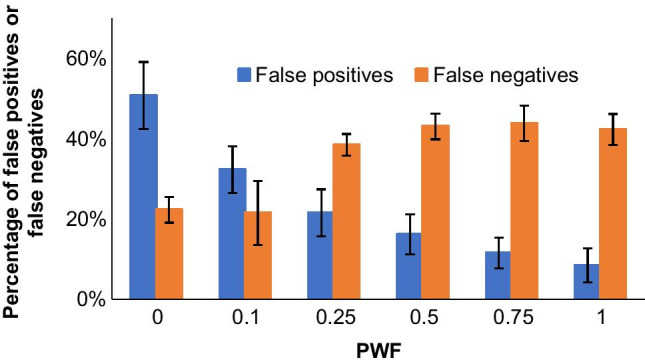
Rate of false negatives and false positives as a function of the pure weight factor (PFW) for the 50 ng/mL spiked mussel extract (10 ng/g dw referring to sample). Match factor cutoff set to allow a 5% of false negatives of the 500 ng/mL level (100 ng/g dw referring to sample) (see Table 2)

As a final optimization step, the most critical parameter in terms of false positives and negatives was optimized at a lower concentration level. Hence, the samples spiked at 50 ng/mL referred to extract (10 ng/g dw referred to sample) were processed with different PWF values using the corresponding match factor cutoff for 5% of false negatives set at the highest spiked level (see above). A summary of false negatives (not detected) and positives (compounds that are incorrectly identified by the software) obtained during this final optimization step is displayed in Fig. 1. As it can be observed, the number of false positives decreases as the PWF moves from a pure reverse (PWF = 0) to a purely forward search (PWF = 1). Conversely, the false negatives rate, which is now higher than 5% due to the lowest spiked concentration, followed the opposite pattern, with PWF 0 and 0.1 leading to the lowest number of false negatives and PWF values in the 0.25–1 range leading to a higher rate of false negatives. Therefore, as a compromise, a PWF of 0.1, which provides the best balance between false positives and negatives at lower concentration levels, was selected as optimal. Although this would result on an expectable rate of false positives of ca. 30%, this value is in practice far lower when the RI matching is implemented.

### Method performance

The proposed method performance was investigated with 17 compounds that were detected in the samples (see “Application to passive sampler and mussel samples”) at two concentration levels as explained in “Material and methods.” For some compounds found in the non-spiked samples at comparatively high concentration levels (3 and 5 compounds for MSPD methods A and B, respectively), the detection frequency could not be calculated in some cases (Table S5). As shown in Table S5, the detection frequency was 100% for most of the compounds at the two concentration levels for the four protocols. However, in the case of passive samplers, lower detection frequencies were observed for the most hydrophobic compounds (i.e., 2-ethylhexyl salicylate, triclosan, 2-ethylhexyl-4-methoxycinnamate, and pyrene) using POCIS. On the other hand, 2,6-di-tert-butyl-4-methylphenol (BHT) and triclosan presented lower detection frequencies in mussel samples likely due to the degradation with sulfuric acid in method A. From such data, SDLs were derived (Table 3).

**Table 3:** Screening detection limit (SDL) of the selected compounds

Name	Screening detection limit (SDL)			
	PDMS (ng)	POCIS (ng)	Method A (ng/g dw)	Method B (ng/g dw)
4-Methylphenol	5	2.5	2	nd
Benzothiazole	5	2.5	2	1
2,6-Di-tert-butyl-1,4-benzoquinone (BHT-Q)	5	2.5	nd	1
2-Ethylhexyl salicylate	5	25	2	10
2,6-Di-tert-butyl-4-methylphenol (BHT)	5	2.5	20	1
Diethyl phthalate	5	2.5	2	1
Benzophenone	5	2.5	nd	1
Tri-n-butyl phosphate (TnBP)	5	2.5	2	1
Benzenesulfonamide	5	2.5	2	nd
Phenanthrene	5	2.5	2	1
Tris(1-chloro-2-propyl) phosphate (TCPP)	5	2.5	2	1
Galaxolide	5	2.5	2	1
Di-iso-butyl phthalate	5	2.5	nd	nd
Di-n-butyl phthalate	5	2.5	2	nd
Triclosan	5	nd	nd	1
Pyrene	5	25	2	1
2-Ethylhexyl 4-methoxycinnamate (EHMC)	5	nd	2	1
2-Ethylhexyl salicylate, TMS	na	25	na	1
Triclosan, TMS	na	2.5	na	1

*na* not analyzed; *nd* not determined due to high concentration in real samples

The SDL for passive samplers was determined to be 5 ng for PDMS (the lowest level tested). In the case of the POCIS, this SDL was 2.5 ng (the lowest level tested) for most compounds, except for 2-ethylhexyl salicylate and pyrene (SDL: 25 ng), where triclosan could only be identified in the derivatized samples. Considering typical values of sampling rate (*R*_s_) ranging between 0.01 and 1 L/day [42–45], that would translate into SDLs referring to water in the 0.18–35 ng/L range, depending on the actual *R*_s_ value and deployment time of the passive sampler (1 or 2 weeks). Reported SDL values in screening studies of surface waters, most of them based on LC-HRMS, were in the range 1.25–500 ng/L [46–48]. As regards mussel samples, not all chemicals’ SDL values could be calculated by both methods, either because of their degradation under the acidic treatment of method A (which targeted more stable compounds) or relatively high levels in the non-spiked sample. Otherwise, SDL values of 1 or 2 ng/g dw (considering an average humidity of 85% that would be equivalent to 0.15–0.30 ng/g wet weight (ww)) could be achieved for all the compounds using at least one of the proposed methods (Table 3). In the literature, SDL values can be found for vegetables, fruits [49–51], and feed but only few studies consider biota samples (fish) with SDL values of 5 ng/g ww [48].

Besides SDLs, recoveries and limits of quantification were also calculated (Table 4) in order to, later on, estimate the concentrations for those 17 chemicals whose standards were available in the environmental samples. The passive sampling and MSPD method used was the one where the chemicals were more often detected. Good absolute recoveries, between 82 and 107% in mussel samples and between 80 and 108% in passive samplers, and precision (RSD values < 9%) were obtained for most compounds (Table 4). LOQs in passive samplers ranged between 0.6 and 3.2 ng. Considering the above mentioned *R*_s_ values, when referring to real samples, LOQs would range between 0.04 and 46 ng/L. In the case of mussel samples, LOQs were in the 0.1–1.8 ng/g dw (equivalent to ca. 0.015–0.27 ng/g ww) range.

**Table 4:** Recoveries and repeatability of the extraction procedures at a concentration level of 50 ng/mL referred to extract. In passive samplers, this is equivalent to 50 ng and 25 ng in PDMS and POCIS, respectively. In mussel samples, this concentration is equivalent to 20 ng/g dw and 10 ng/g dw mussel, for methods A and B, respectively

Name	Passive sampler				MSPD			
	Method	Recovery (%)	Repeatability (RSD %)	LOQs (ng)	Method	Recovery (%)	Repeatability (RSD %)	LOQs (ng/g)
4-Methylphenol	POCIS	108	4	2.4	B	nd	nd	1.0
Benzothiazole	POCIS	99	2	2.1	B	98	1	0.7
2,6-Di-tert-butyl-1,4-benzoquinone (BHT-Q)	PDMS	102	1	2.0	A	nd	nd	1.3
2-Ethylhexyl salicylate	PDMS	102.6	0.7	2.2	A	104	2	1.8
2,6-Di-tert-butyl-4-methylphenol (BHT)	PDMS	103	2	2.2	B	105	3	0.8
Diethyl phthalate	PDMS	106.1	0.9	1.4	B	85	2	0.7
Benzophenone	PDMS	95	1	3.2	B	82	2	1.0
Tri-n-butyl phosphate (TnBP)	PDMS/POCIS	99/ 106.2	9/0.6	1.5/2.4	B	107.2	0.7	0.7
Benzenesulfonamide	POCIS	90	3	1.2	B	nd	nd	0.9
Phenanthrene	POCIS	80	6	2.1	A	94	3	1.8
Tris(1-chloro-2-propyl) phosphate (TCPP)	POCIS	100	1	1.3	B	99.5	0.9	0.7
Galaxolide	PDMS	97	1	2.3	B	89	3	0.6
Di-iso-butyl phthalate	PDMS/POCIS	99/98	2/1	1.2/1.1	B	nd	nd	0.1
Di-n-butyl phthalate	PDMS/POCIS	97/98	5/4	0.6/1.2	B	nd	nd	0.4
Triclosan	POCIS	105	1	1.0	B	100	1	0.5
Pyrene	PDMS	99.6	0.7	2.0	A	102.7	0.5	0.5
2-Ethylhexyl 4-methoxycinnamate (EHMC)	PDMS	98	2	1.9	B	103	1	0.7

*nd* not determined due to high concentration in real samples

### Application to passive sampler and mussel samples

**Fig. 2:**
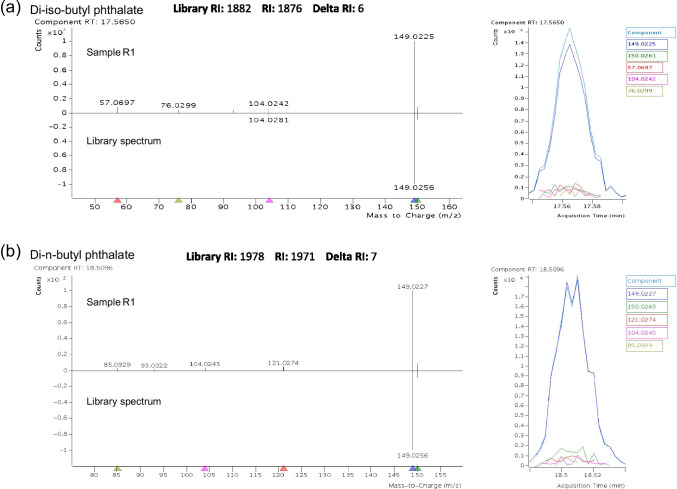
Deconvoluted spectra and chromatograms of **a** di-iso-butyl phthalate, and **b** di-n-butyl phthalate

The optimized screening method was applied to samples from the marine environment and river water from Galicia (NW Spain), including mussels and passive samplers. A total of 75 compounds were identified in these samples (summarized in Table 5, further details on each particular sample and compound usage is presented in Table S6), 46 of which were identified only in the passive samplers and 52 only in mussels, while 23 could be positively detected in both water and mussel samples. The use of RI allowed the identification of isomers or compounds with similar spectra, e.g., di-iso-butyl phthalate and di-n-butyl phthalate (Fig. 2). Further examples of some of deconvoluted spectra as compared to the libraries’ spectra are presented in Figure S5.

**Table 5:** List of compounds detected in the real samples. Further details are provided in Table S6

Compounds detected		
1,1′-Biphenyl	Anthracene	Musk ambrette (natural)
1,2,3-Trichlorobenzene	Benzenesulfonamide	Naphthalene
1,2,4-Trichlorobenzene	Benzophenone	Naproxen
1,6-Diisopropylnapthalene	Benzothiazole	Octocrylene
1-Naphthol	Benzyl butyl phthalate	Oxybenzone (BP-3)
2-(Methylthio)benzothiazole	Bis(2-ehtylhexyl) adipate	Paraxanthine
2,2',3,4,4',5'-Hexachlorobiphenyl (PCB 118)	Bis(2-ethylhexyl) phthalate	Phenanthrene
2,2',4,4',5,5'-Hexachlorobiphenyl (PCB 151)	Bisphenol A	Phenol
2,2',4,4'-Tetrabromodiphenyl ether (BDE 47)	Bornyl acetate	Phthalide
2,2',4,5,5'-Pentachlorobiphenyl (PCB 101)	Butylated hydroxyanisole (BHA)	Phthalimide
2,6-Dibromophenol	Caffeine	Pyrene
2,6-Diisopropylnapthalene	Camphor	Theobromine
2,6-Di-tert-butyl-1,4-benzoquinone (BHT-Q)	DEET/diethyltoluamide	Thymol
2,6-Di-tert-butyl-4-methylphenol (BHT)	Di-(2-ethylhexyl) terephthalate	Tolytriazole
2-Aminobenzothiazol	Dicyclohexyl phthalate	Tonalide
2-Ethylhexyl 4-methoxycinnamate (EHMC)	Diethyl phthalate	Triclosan
2-Ethylhexyl diphenyl phosphate (EHDPP)	Di-iso-butyl phthalate	Tri-iso-butyl phosphate (TiBP)
2-Ethylhexyl salicylate	Dimethyl phthalate	Tri-n-butyl phosphate (TnBP)
2-Methylnaphthalene	Di-n-butyl phthalate	Triphenyl phosphate
2-Methylphenol	Diphenyl ether	Tris(1-chloro-2-propyl) phosphate (TCPP)
3,4-DCA/3,4-dichloroaniline	Galaxolide	Tris(2-butoxyethyl) phosphate (TBEP)
3,5-Di-tert-butyl-4-hydroxybenzaldehyde (BHT-CHO)	Ibuprofen	Venlafaxine
4-Chlorophenol	Indole	α-Hexachlorocyclohexane (α-HCH)
4-Hydroxybenzoic acid	Ketoprofen	α-Methylstyrene
4-Methylphenol	Metolcarb	α-Terpineol

**Fig. 3:**
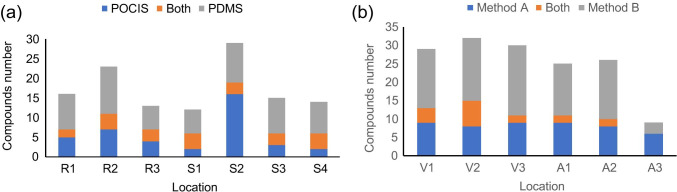
Distribution of the identified compounds **a** in the water samples according to the passive sampler and **b** in mussel samples according to the MSPD method

By sample (Fig. 3a), the number of compounds detected in passive samplers was in the range of 12 to 29, without clear differences between estuary and river water. This may be due to the longer deployment time of the passive samplers in the marine environment (1 vs 2 weeks in freshwater and marine water, respectively), which was performed as to compensate for the expectably lower concentrations. R2, in river, and S2, in estuarine, samples, which are the sampling points closer to the discharge of wastewater treatment plants (WWTPs), were the most polluted samples in terms of detected chemicals, with 23 and 29 identified compounds, respectively. As regards mussel samples (Fig. 3b), the number of identified compounds ranged from 9 to 32, being this number quite stable in Vigo estuary (29–32 compounds at each location) but more variable between different locations of Arousa estuary (9–26 compounds), where the sampling point A3 was far cleaner (Fig. 3b).

Regarding sampling mode, more compounds per sample were detected in PDMS than in POCIS, with 2–4 compounds detected in both samplers, except in Sample S2 where more compounds were identified in the POCIS (Fig. 3a). The different patterns in this sample are mostly due to the fact that polar compounds related with the proximity to a WWTP, such as the silylated derivatives of ibuprofen, naproxen, and ketoprofen or dimethyl phthalate, were detected here. Thus, these results show the complementarity of these two passive sampling techniques for the detection of contaminants. In mussel samples, the number of compounds detected by MSPD method A was lower than by method B, with 0–7 compounds detected by both methods, but for the cleanest sample, A3, where more compounds were detected by method A (Fig. 3b).

By library, 70% of the compounds detected in the passive samplers were non-derivatized analytes from the in-house library (55% in POCIS and 82% in PDMS), 11% from the pesticides library (21% in POCIS and 9% in PDMS), 13% derivatized compounds from the in-house library (21% in POCIS and 9% in PDMS), and the rest (7%) from the two libraries (Figure S4b). A similar distribution was found in mussel samples, i.e., 77% from non-derivatized analytes (in-house library), 12% from pesticides library, 4% of derivatized compounds (in-house library), and 8% from two of the libraries (Figure S4a). These findings highlight the relevance of expanding high-resolution EI-MS libraries.

Plasticizers were the major contaminants detected in all the environmental samples, which collectively accounted for 25–38% of the total number of contaminants for each matrix. In particular, 4 plasticizers, i.e., dicyclohexyl phthalate, di-iso-butyl phthalate, diethylphthalate, and di-n-butyl phthalate, were the compounds more frequently detected in all samples (> 85% samples). However, in water, the type of compounds detected in each passive sampler differs. While in POCIS, pharmaceuticals represent a 17% of the total number of compounds, being the second class of pollutants, and pesticides the third-class with a 14%; in PDMS, UV filters, fragrances, and pesticides represent the second class of chemicals with a 12% detection rate each. In mussels, 83% of the compounds detected belong to one of the following classes: plasticizers (23%), industrial chemicals (23%), pesticides (17%), PAHs (10%), and fragrances (10%). The type of compounds detected also differs by the MSPD method used. From the most frequently detected families, plasticizers are detected by both methods, pesticides and fragrances are mainly detected in MSPD method B extracts, while PAHs in MSPD method A extracts. On the other hand, industrial chemicals, which include compounds with different chemical characteristics, are detected, depending on the structure, by method A (e.g., trichlorobenzenes) or by method B (e.g., phenols). Finally, it is clear that mussels and PDMS can capture more hydrophobic chemicals as compared to POCIS, which are designed to detect more polar analytes.

### Estimated concentrations

The concentrations (Table 6) of the 17 compounds detected in the samples, which could be validated (see “Method performance”), were calculated using the method selected in Table 4. In passive samplers, different profiles were observed. Thus, on the one hand, some compounds (e.g., diethyl phthalate and galaxolide) were found at higher concentration in passive samplers close to sewage discharges, pointing to wastewater treatment plants as their main source, whereas, for instance, on the other hand, EHMC was detected only in marine samples. Considering the typical values of sampling rate (*R*_s_) of 0.01 and 1 L/day [42–45], the amounts measured would represent concentrations in the sub-ng/L to low µg/L level.

**Table 6:** Estimated mass/concentration of some of the detected analytes in the passive samplers (ng) and mussels (ng/g); *nd* means not detected (< LOD). For sample codes, please refer to the “Materials and methods—Sampling and deployment of passive samplers” section and Figure S1

Compound	Passive sampler (ng)							Mussel (ng/g)					
	S1	S2	S3	S4	R1	R2	R3	V1	V2	V3	A1	A2	A3
4-Methylphenol	nd	nd	nd	nd	3	nd	nd	6	6	11	4	3	< LOQ
Benzothiazole	nd	2	nd	nd	nd	nd	nd	6	nd	nd	nd	nd	< LOQ
2,6-Di-tert-butyl-1,4-benzoquinone (BHT-Q)	nd	6	nd	nd	< LOQ	2	< LOQ	18	9	14	8	10	6
2-Ethylhexyl salicylate	nd	< LOQ	< LOQ	< LOQ	nd	nd	nd	nd	14	nd	7	nd	nd
2,6-Di-tert-butyl-4-methylphenol (BHT)	nd	nd	nd	nd	4	136	19	nd	nd	7		nd	nd
Diethyl phthalate	17	85	35	48	496	2188	nd	1208	549	165	288	492	nd
Benzophenone	nd	nd	6	nd	nd	nd	nd	nd	nd	28	nd	nd	nd
Tri-n-butyl phosphate (TnBP)	nd	28	nd	nd	nd	70	19	nd	30	nd	nd	nd	nd
Benzenesulfonamide	nd	20	nd	nd	nd	nd	nd	76	43	117	114	92	nd
Phenanthrene	nd	431	nd	nd	32	39	71	6	nd	nd	3	124	nd
Tris(1-chloro-2-propyl) phosphate (TCPP)	nd	78	nd	nd	nd	15	8	5	1	6	< LOQ	7	nd
Galaxolide	47	65	nd	39	501	813	207	nd	nd	48	36	23	nd
Di-iso-butyl phthalate	68	95	78	73	< LOQ	6	39	260	138	295	252	256	nd
Di-n-butyl phthalate	67	157	72	74	13	29	44	293	2746	571	461	392	nd
Triclosan	nd	2	nd	nd	nd	nd	nd	nd	nd	15	9	nd	nd
Pyrene	nd	7	nd	nd	nd	nd	nd	4	7	nd	4	11	6
2-Ethylhexyl 4-methoxycinnamate (EHMC)	195	nd	245	248	nd	nd	nd	1570	711	nd	nd	nd	nd

In mussel samples, the plasticizers diethyl phthalate and di-n-butyl phthalate and the UV filter EHMC were the compounds found at higher concentrations; up to 2746 ng/g dw for di-n-butyl phthalate (equivalent to 412 ng/g ww).

## Conclusions

We have optimized the different parameters used for the GC-EI-HRMS screening of organic contaminants by the Agilent Unknowns Analysis SureMass algorithm, finding out that the selection of mass tolerance and weighting of reversed-/forward-searching plays a relevant role in the potential number of false positives and false negatives. Furthermore, an HRMS library was created, where the inclusion of RIs further provides confidence on the identification. The optimized method provides good SDL and was able to successfully identify 75 chemicals in marine and freshwater samples.

## Supplementary Information

Below is the link to the electronic supplementary material.

Supplementary file1 (PDF 2406 KB)
