# Supplementary material for: Development and application of an in-house library and workflow for gas chromatography–electron ionization–accurate-mass/high-resolution mass spectrometry screening of environmental samples
Source: Anal Bioanal Chem. 2021 Dec 4;414(21):6327–40. doi: 10.1007/s00216-021-03810-w (PMC9372009; doi:10.1007/s00216-021-03810-w)
Supplement: Supplementary file 1 — Supplementary file1 (PDF 2406 KB) [file 216_2021_3810_MOESM1_ESM.pdf]

SUPPORTING INFORMATION TO:

Development and application of an in-house library and workflow for gas chromatography-electron ionization accurate-mass/high-resolution-mass spectrometry screening of environmental samples

Verónica Castro[1], José Benito Quintana[1,*], Javier López-Vázquez[1], Nieves Carro[2], Julio Cobas[2], Denis Bilbao[3,4], Rafael Cela[1], Rosario Rodil[1,*]

[1] Department of Analytical Chemistry, Institute of Research on Chemical and Biological Analysis (IAQBUS), Universidade de Santiago de Compostela, 15782 Santiago de Compostela, Spain.

[2] INTECMAR - Technological Institute for the Monitoring of the Marine Environment of Galicia, Peirao de Vilaxoán S/N, 36611, Vilagarcía de Arousa, Spain

[3] Department of Analytical Chemistry, University of the Basque Country (UPV/EHU), Leioa Basque Country, 48940, Spain

[4] Research Centre for Experimental Marine Biology and Biotechnology, University of the Basque Country (PiE-UPV/EHU), Plentzia Basque Country, 48620, Spain

*Corresponding authors: jb.quintana@usc.es (J.B. Quintana) rosario.rodil@usc.es (R. Rodil)

INDEX:

Table S1: Model compounds used during Unknowns Analysis method optimization.

Table S2: Compounds included in the in-house accurate mass library where a non-derivatized spectra was recorded

Table S3: Compounds included in the In-house library that could be derivatized, where TMS indicates that the compound is derivatized with a trimethylsilyl group, and 2TMS and 3TMS with two or three trimethylsilyl groups, respectively.

Table S4: List of chemicals included in the modified commercial Agilent RTL Pesticide Library.

Table S5: Detection frequency of the compounds selected for method validation, when spiked at two concentration/mass levels.

Table S6: Compounds found in passive sampler and mussel samples. Coding: 0: not detected; 1: detected; 1S: detected as silylated derivative.

Figure S1: Location of the samples analyzed in this work.

Figure S2: Percentage of false negatives as a function of the accurate-mass tolerance (AMT) for different values of pure weight factor (PWF). Mussel spiked concentration: 100 ng/d dw, equivalent to 500 ng/g in the extract.

Figure S3: Match factors obtained for the model analytes at different values of pure weight factor (PWF). Mussel spiked concentration: 100 ng/d dw, equivalent to 500 ng/g in the extract.

Figure S4: Distribution of compounds detected in the samples according to the library used, (a) for mussel samples; (b) in passive samplers.

Figure S5: Examples of chemicals detected in the samples.

Table S1: Model compounds used during Unknowns Analysis method optimization.

Name	Formula	CAS No.	t_R (min)
1,1'-(2,2-dichloroethenylidene)bis[4-chloro-benzene] (p,p'-DDE)	C14H8Cl4	72-55-9	20.06
1,1,1-Trichloro-2,2-bis(4-chlorophenyl)ethane (p,p'-DDT)	C14H9Cl5	50-29-3	21.50
2,3',4',6-Tetrabromodiphenyl ether (BDE 71)	C12H6Br4O	189084-62-6	23.23
2,4,4′-Tribromodiphenyl ether (BDE 28)	C12H27Br3O	41318-75-6	21.33
2,4,6-Trichlorobiphenyl (PCB 30)	C12H7Cl3	35693-92-6	16.48
2-Bromophenol	C6H5BrO	95-56-7	7.59
2-Chlorophenol	C6H5ClO	95-57-8	6.32
Anthracene	C14H10	120-12-7	16.95
Benzo[a]pyrene	C20H12	50-32-8	26.25
Benzophenone	C13H10O	119-61-9	15.25
Benzophenone-3 (BP-3)	C14H12O3	131-57-7	19.32
Benzothiazole	C7H5NS	95-16-9	9.93
Bis(2-ethylhexyl) phthalate	C24H3804	117-81-7	23.53
Butylated hydroxyanisole (BHA)	C11H16O2	25013-16-5	13.26
Butylated hydroxytoluene (BHT)	C15H24O	128-37-0	13.61
Di-n-butyl phthalate	C16H2204	84-74-2	18.50
Galaxolide	C18H26O	1222-05-5	17.50
Methylparaben	C8H8O3	99-76-3	13.61
Octocrylene	C24H27NO2	6197-30-4	24.53
Propylparaben	C10H1203	94-13-3	15.57
Pyrene	C16H10	129-00-0	20.13
Tonalide	C18H26O	21145-77-7	17.61
Triclosan	C12H7Cl3O2	3380-34-5	19.99
Tri-n-butyl phosphate (TnBP)	C12H27O4P	126-73-8	15.18
Triphenyl phosphate (TPhP)	C18H15O4P	115-86-6	22.47
α-Hexachlorocyclohexane (α-HCH)	C6H6Cl6	319-84-6	16.58

Table S2: Compounds included in the in-house accurate mass library where a non-derivatized spectra was recorded

Name	Formula	CAS No.	Retention Index
(2,4'-Dichlorodiphenyl)dichloroethane (2,4'-DDD)	C14H10Cl4	53-19-0	2213
1,1,1-Trichloro-2-(2-chlorophenyl)-2-(4-chlorophenyl)ethane (2,4'-DDT)	C14H9Cl5	789-02-6	2286
1,1,1-Trichloro-2,2-bis(4-chlorophenyl)ethane (4,4'-DDT)	C14H9Cl5	50-29-3	2367
1,1'-Biphenyl	C12H10	92-52-4	1390
1,1-Dichloro-2,2-bis(4-chlorophenyl)ethane (4,4'-DDD)	C14H10Cl4	72-54-8	2292
1,2,3,4-Tetrachlorobenzene	C6H2Cl4	634-66-2	1392
1,2,3-Trichlorobenzene	C6H3Cl3	87-61-6	1230
1,2,4,5-Tetrachlorobenzene	C6H2Cl4	95-94-3	1343
1,2,4-Trichlorobenzene	C6H3Cl3	120-82-1	1192
1,3,5-Tribromobenzene	C6H3Br3	626-39-1	1440
1,4-Cineole	C10H18O	470-67-7	1017
11-Hydroxy- Δ9-tetrahydrocannabinol (11-OH-THC)	C21H30O3	36557-05-8	2888
17a-Ethinylestradiol	C20H24O2	57-63-6	2766
1-Fluoronaphthalene	C10H7F	321-38-0	1195
1H-Benzotriazole	C6H5N3	95-14-7	1505
1-Methylnaphthalene	C11H10	90-12-0	1308
2-(2-Chlorophenyl)-2-(4-chlorophenyl)-1,1-dichloroethene (2,4'-DDE)	C14H8Cl4	3424-82-6	2129
2-(Methylthio)benzothiazole	C8H7NS2	615-22-5	1629
2,2',3,4,4',5,5'-Heptachlorobiphenyl (PCB 180)	C12H3Cl7	35065-29-3	2516
2,2',3,4,4',5'-Hexachlorobiphenyl (PCB 138)	C12H4Cl6	35065-28-2	2366
2,2',3,4,4'-Pentabromodiphenyl ether (BDE 85)	C12H5Br5O	182346-21-0	2777
2,2',4,4',5,5'-Hexachlorobiphenyl (PCB 153)	C12H4Cl6	35065-27-1	2303
2,2',4,4',5,6'-Hexabromodiphenyl ether (BDE 154)	C12H4Br6O	207122-15-4	3190
2,2',4,4',5-Pentabromodiphenyl ether (BDE 99)	C12H5Br5O	60348-60-9	2835
2,2',4,4'-Tetrabromodiphenyl ether (BDE 47)	C12H6Br4O	5436-43-1	2541
2,2',4,5,5'-Pentachlorobiphenyl (PCB 101)	C12H5Cl5	37680-73-2	2123
2,2',4,5',6-Pentabromobiphenyl (PBB 103)	C12H5Br5	59080-39-6	2538
2,2',5,5'-Tetrachlorobiphenyl (PCB 52)	C12H6Cl4	35693-99-3	1945
2,2-Bis(4-chlorophenyl)-1,1-dichloroethylene (4,4'-DDE)	C14H8Cl4	72-55-9	2183
2,2-Dimethoxy-2-phenylacetophenone	C16H16O3	24650-42-8	1896
2,3,3',4,4',5-Hexachlorobiphenyl (PCB 156)	C12H4Cl6	38380-08-4	2480
2,3,3',4,4'-Pentachlorobiphenyl (PCB 105)	C12H5Cl5	32598-14-4	2315
2,3',4,4',5-Pentachlorobiphenyl (PCB 118)	C12H5Cl5	31508-00-6	2257
2,3,4,5,6-Pentachlorotoluene	C7H3Cl5	877-11-2	1694
2,3',4',6-Tetrabromodiphenyl ether (BDE 71)	C12H6Br4O	189084-62-6	2520
2,3',4',6-Tetrachlorobiphenyl (PCB 71)	C12H6Cl4	52663-58-8	1978
2,3,4-Trichlorophenol	C6H3Cl3O	15950-66-0	1389
2,3,5,6-Tetrachlorophenol	C6H2Cl4O	935-95-5	1570
2,3,5-Trichlorophenol	C6H3Cl3O	933-78-8	1358
2,3,6-Trichlorophenol	C6H3Cl3O	933-75-5	1395
2,3,6-Trichlorotoluene	C7H5Cl3	2077-46-5	1335
2,3-Dichlorophenol	C6H4Cl2O	576-24-9	1193

Name	Formula	CAS No.	Retention Index
2,3-Dichlorotoluene	C7H6Cl2	32768-54-0	1127
2,4,4'-Tribromodiphenyl ether (BDE 28)	C12H27Br3O	488710-19-6	2286
2,4,4'-Trichlorobiphenyl (PCB 28)	C12H7Cl3	7012-37-5	1878
2,4,5-Trichloroaniline	C6H4Cl3N	636-30-6	1584
2,4',5-Trichlorobiphenyl (PCB 31)	C12H7Cl3	16606-02-3	1874
2,4,5-Trichlorotoluene	C7H5Cl3	6639-30-1	1308
2,4,6-Trichlorobiphenyl (PCB 30)	C12H7Cl3	35693-92-6	1768
2,4,6-Trichlorophenol	C9H9Cl3O	88-06-2	1372
2,4-Dichlorophenol	C6H4Cl2O	120-83-2	1184
2,4-Dinitrotoluene	C7H6N2O4	121-14-2	1570
2,5-Dichlorophenol	C6H4Cl2O	583-78-8	1179
2,5-Dichlorotoluene	C7H6Cl2	19398-61-9	1126
2,6-Dibromophenol	C6H4Br2O	608-33-3	1397
2,6-Dichlorophenol	C6H4Cl2O	87-65-0	1211
2,6-Dichlorotoluene	C7H6Cl2	118-69-4	1129
2,6-Diisopropylnapthalene	C16H20	24157-81-1	1736
2,6-Dinitrotoluene	C7H6N2O4	606-20-2	1489
2,6-Di-tert-butyl-1,4-benzoquinone (BHT-Q)	C14H20O2	719-22-2	1471
2,6-Di-tert-butyl-4-hydroxy-4-methyl-2,5-cyclohexadien-1-one (BHT-OH)	C15H24O2	10396-80-2	1791
2,6-Di-tert-butyl-4-methylphenol (BHT)	C15H24O	128-37-0	1521
2-Aminobenzothiazole	C7H6N2S	136-95-8	1695
2-Bromophenol	C6H5BrO	95-56-7	1082
2-Chlorophenol	C6H5ClO	95-57-8	994
2-Chlorotoluene	C7H7Cl	95-49-8	959
2-Ethylhexyl 4-(dimethylamino)benzoate (ODPABA)	C17H27NO2	58817-05-3	2284
2-Ethylhexyl 4-methoxycinnamate (EHMC)	C18H26O3	5466-77-3	2331
2-Ethylhexyl diphenyl phosphate (EHDPP)	C20H27O4P	1241-94-7	2447
2-Ethylhexyl salicylate	C15H22O3	118-60-5	1815
2-Ethylhexyl-4-(dimethylamino)benzoate	C17H27NO2	21245-02-3	2286
2-Isopropylthioxanthone	C16H14OS	5495-84-1	2410
2-Methylbenzothiazole	C8H7NS	120-75-2	1316
2-Methylnaphthalene	C11H10	91-57-6	1324
2-tert-Butyl-1,4-dihydroxybenzene (TBHQ)	C10H14O2	1948-33-0	1589
3,4-Dichlorophenol	C6H4Cl2O	95-77-2	1435
3,4-Dichlorotoluene	C7H6Cl2	95-75-0	1154
3,4-Methylenedioxymethamphetamine (MDMA)	C11H15NO2	42542-10-9	1553
3,4-Methylenedioxy-N-ethylamphetamine (MDEA)	C12H17NO2	82801-81-8	1599
3,5-Dichlorophenol	C6H4Cl2O	591-35-5	1410
3,5-Di-tert-butyl-4-hydroxybenzaldehyde (BHT-CHO)	C15H22O2	1620-98-0	1780
3,5-Di-tert-butyl-4-hydroxybenzoic acid (BHT-COOH)	C15H23NO2	1421-49-4	1699
3-Chlorotoluene	C7H7Cl	108-41-8	957
4-Acetaminoantipyrine	C13H15N3O2	83-15-8	2317
4-Chloro-2-methylaniline	C7H8ClN	95-69-2	1332
4-Chloroaniline	C6H6ClN	106-47-8	1252
4-Chlorophenol	C6H5ClO	106-48-9	1277

Name	Formula	CAS No.	Retention Index
4-Chlorotoluene	C7H7Cl	106-43-4	964
4-Formylaminoantipyrine	C12H13N3O2	1672-58-8	1992
4-Hydroxybenzoic acid propyl ester	C10H1203	94-13-3	1689
4-Methyl-1H-benzotriazole	C7H7N3	29878-31-7	1618
4-Methylbenzophenone	C14H12O	134-84-9	1773
4-Nonylphenol	C15H24O	104-40-5	1885
4-Octylphenol	C14H22O	1806-26-4	1781
4-Phenylbenzophenone	C19H14O	2128-93-0	2487
5-Chlorobenzotriazole	C6H4ClN3	94-97-3	1799
5-Methyl-1H-benzotriazole	C7H7N3	136-85-6	1572
Acenaphthene	C12H10	83-32-9	1495
Acenaphthylene	C12H8	208-96-8	1464
a-Hexachlorocyclohexane (a-HCH)	C6H6Cl6	319-84-6	1783
Alprazolam	C17H13ClN4	28981-97-7	3063
a-Methylstyrene	C9H10	98-83-9	986
Anthracene	C14H10	120-12-7	1802
Antipyrine	C11H12N2O	60-80-0	1903
a-Pinene	C10H16	80-56-8	935
a-Terpineol	C10H18O	98-55-5	1196
Avobenzone	C20H22O3	70356-09-1	2830
Benz[a]anthracene	C18H12	56-55-3	2468
Benzenesulfonamide	C6H7NO2S	98-10-2	1809
Benzo[a]pyrene	C20H12	50-32-8	2889
Benzo[b]fluoranthene	C20H12	205-99-2	2792
Benzo[ghi]perylene	C22H12	191-24-2	3257
Benzo[k]fluoranthene	C20H12	207-08-9	2804
Benzophenone	C13H10O	119-61-9	1644
Benzothiazole	C7H5NS	95-16-9	1244
Benzyl 4-hydroxybenzoate	C14H12O3	94-18-8	2200
Benzyl butyl phthalate	C19H20O4	85-68-7	2373
Benzyl salicylate	C14H12O3	118-58-1	1895
Bis(2-ehtylhexyl) adipate	C22H42O4	103-23-1	2408
Bis(2-ethylhexyl) phthalate	C24H38O4	117-81-7	2559
Bis(2-methoxyethyl) phthalate	C14H18O6	117-82-8	2022
Bisphenol A	C15H16O2	80-05-7	2231
Bisphenol A diacetate	C19H20O4	10192-62-8	2378
Bisphenol AF	C15H10F6O2	1478-61-1	2095
Bisphenol B	C16H18O2	77-40-7	2330
Bisphenol E	C14H14O2	66-32-8	2178
Bisphenol F	C13H12O2	2467-02-9	2124
Borneol	C10H18O	507-70-0	1172
Bornyl acetate	C12H20O2	76-49-3	1290
Butyl 4-hydroxybenzoate	C11H14O3	94-26-8	1758
Butylated hydroxyanisole (BHA)	C11H16O2	121-00-6	1507
Caffeine	C8H10N4O2	58-08-2	1862

Name	Formula	CAS No.	Retention Index
Camphene	C10H16	79-92-5	950
Camphor	C10H16O	76-22-2	1149
Cannabidiol	C21H30O2	13956-29-1	2439
Carvacrol	C10H14O	499-75-2	1323
Cashmeran	C14H22O	33704-61-9	1509
Celestolide	C17H24O	13171-00-1	1728
Chlordiazepoxide	C16H14ClN3O	58-25-3	2599
Chlorfenvinphos	C12H14Cl3O4P	470-90-6	2096
Chlorpyrifos	C9H11Cl3NO3PS	2921-88-2	2002
Chrysene	C18H12	218-01-9	2481
Citalopram	C20H21FN2O	59729-33-8	2475
Clozapine	C18H19ClN4	5786-21-0	2973
Cocaethylene	C18H23NO4	529-38-4	2320
Cocaine	C17H21NO4	50-36-2	2261
Cotinine	C10H12N2O	486-56-6	1722
Cresyl diphenyl phosphate (DCP)	C19H17O4P	26444-49-5	2510
Di-(2-ethylhexyl) terephthalate	C24H38O4	6422-86-2	2762
Diamyl phthalate	C18H26O4	131-18-0	2167
Diazepam	C16H13ClN2O	439-14-5	2521
Dibenz[ah]anthracene	C22H14	53-70-3	3206
Di-n-butyl phthalate	C16H22O4	84-74-2	1978
Dicyclohexyl phthalate	C20H26O4	84-61-7	2552
Diethyl phthalate	C12H14O4	84-66-2	1624
Di-iso-butyl phthalate	C16H22O4	84-69-5	1882
Di-iso-decyl phthalate	C28H46O4	89-16-7	2938
Di-iso-nonyl cyclohexane-1,2-dicarboxylate (DINCH)	C26H48O4	166412-78-8	2769
Di-iso-nonyl phthalate	C26H42O4	20548-62-3	2813
Dimethyl phthalate	C10H10O4	131-11-3	1466
Dimethyl terephthalate	C10H10O4	120-61-6	1546
Di-n-octyl phthalate	C24H38O4	117-84-0	2750
Diphenyl ether	C12H10O	101-84-8	1419
Diphenyl phosphate	C12H11O4P	838-85-7	988
E-Diethylstilbestrol	C18H20O2	56-53-1	2372
Estrone	C18H22O2	53-16-7	2683
Ethyl 4-hydroxybenzoate	C9H10O3	120-47-8	1557
Ethyl-4-dimethylaminobenzoate	C11H15NO2	10287-53-3	1732
Ethynylestradiol 3-methyl ether (Mestranol)	C21H26O2	72-33-3	2706
Eucalyptol	C10H18O	470-82-6	1034
Eugenol	C10H12O2	97-53-0	1374
Fentanyl	C22H28N2O	437-38-7	2812
Flunitrazepam	C16H12FN3O3	1622-62-4	2706
Fluoranthene	C16H10	206-44-0	2090
Fluorene	C13H10	86-73-7	1595
Flurazepam	C21H23ClFN3O	17617-23-1	2868
Galaxolide	C18H26O	1222-05-5	1864

Name	Formula	CAS No.	Retention Index
Geraniol	C10H18O	106-24-1	1294
Heroine	C21H23NO5	561-27-3	2723
Hexachlorobenzene	C6Cl6	118-74-1	1716
Indeno[1,2,3-cd]pyrene	C22H12	193-39-5	3195
Indole	C8H7N	120-72-9	1324
Isobutyl 4-hydroxybenzoate	C11H14O3	4247-023	1714
Ketamine	C13H16ClNO	6740-88-1	1896
Linalool	C10H18O	78-70-6	1105
Mephedrone	C11H15NO	1189805-46-6	1473
Methyl 3-chloro-4-hydroxybenzoate	C8H7ClO3	3964-57-6	1473
Methyl 4-hydroxybenzoate	C8H8O3	99-76-3	1491
Methyl-3,5-dibromo-4-hydroxybenzoate	C8H6Br2O3	41727-47-3	1837
Midazolam	C18H13ClFN3	59467-70-8	2685
Mirtazapine	C17H19N3	85650-52-8	2316
Musk ambrette (artificial)	C12H16N2O5	83-66-9	1846
Musk ketone	C14H18N2O5	81-14-1	1995
Musk tibetene	C13H18N2O4	145-39-1	1952
Naphthalene	C10H8	91-20-3	1230
Nicotine	C10H14N2	54-11-5	1366
N-Nitrosodiphenylamine	C12H10N2O	86-30-6	1640
Octocrylene	C24H27NO2	6197-30-4	2692
Oxybenzone (BP-3)	C14H12O3	131-57-7	2048
Paraxanthine	C7H8N4O2	611-59-6	1889
p-Cymene	C10H14	99-87-6	1027
Pentachlorobenzene	C6HCl5	608-93-5	1540
Perylene	C20H12	198-55-0	2937
Phantolide	C17H24O	15323-35-0	1771
Phenanthrene	C14H10	85-01-8	1790
Phenazone (Antipyrine)	C11H12N2O	60-80-0	1920
Piperonyl butoxide	C19H30O5	51-03-6	2426
Prazepam	C19H17ClN2O	2955-38-6	2743
Propyphenazone	C14H18N2O	479-92-5	1983
p-Toluenesulfonamide	C7H9NO2S	70-55-3	1754
Pyrene	C16H10	129-00-0	2128
Sertraline	C17H17NCl2	79617-96-2	2433
ß-Estradiol	C18H24O2	50-28-2	2707
ß-Pinene	C10H16	127-91-3	979
Styrene	C8H8	100-42-5	901
Tetraethyl 1,2-ethanediylbis(phosphonate) (TEEdP)	C10H24O6P2	995-32-4	1880
Tetrahydrocannabinol (THC)	C21H30O2	26-51-4	2546
Theobromine	C7H8N4O2	83-67-0	1879
Thymol	C10H14O	89-83-8	1299
Tolytriazole (Methyl-1H-benzotriazole mixture)	C14H14N6	29385-43-1	1928
Tonalide	C18H26O	21145-77-7	1879
Traseolide	C18H26O	68140-48-7	1863

Name	Formula	CAS No.	Retention Index
Trembolone	C18H22O2	10161-33-8	2790
Tri(2-chloroethyl) phosphate (TCEP)	C6H12Cl3O4P	115-96-8	1782
Triclosan	C12H7Cl3O2	3380-34-5	2132
Triclosan methyl ether	C13H9Cl3O2	1000-76-6	2141
Tri-iso-butyl phosphate (TiBP)	C12H27O4P	126-71-6	1520
Tri-n-butyl phosphate (TnBP)	C12H27O4P	126-73-8	1657
Tripentyl phosphate (TPeP)	C15H33O4P	2528-38-3	1935
Triphenyl phosphate (TPhP)	C18H15O4P	115-86-6	2419
Tris(1,3-dichloroisopropyl) phosphate (TDCP)	C9H15Cl6O4P	13674-87-8	2349
Tris(1-Chloro-2-Propyl) phosphate (TCPP)	C9H18Cl3O4P	13674-84-5	1810
Tris(2,3-dibromopropyl) phosphate (TDBPP)	C9H15Br6O4P	126-72-7	1174
Tris(2-butoxyethyl) phosphate (TBEP)	C18H39O7P	78-51-3	2409
Tris(2-ethylhexyl) phosphate (TEHP)	C24H51O4P	78-42-2	2479
Verbenone	C10H14O	1196-01-6	1215
Z-Diethylstilbestrol	C18H20O2	22610-99-7	2322
Zolpidem	C19H21N3O	82626-48-0	2906
γ-Terpinene	C10H16	99-85-4	1062

Table S3: Compounds included in the In-house library that could be derivatized, where TMS indicates that the compound is derivatized with a trimethylsilyl group, and 2TMS and 3TMS with two or three trimethylsilyl groups, respectively.

Name	Formula	CAS No.*	Retention Index
[(4-Chloro-o-tolyl)oxy]acetic acid, TMS derivative	C12H17ClO3Si	94-74-6	1687
11-Hydroxy-Δ9-tetrahydrocannabinol (11-OH-THC), 2TMS derivative	C27H46O3Si2	36557-05-8	2663
11-nor-9-carboxy-Δ9-Tetrahydrocannnabinol (THC-COOH), 2TMS derivative	C27H44O4Si2	56354-06-4	2801
17a-Ethynyloestradiol, 2TMS derivative	C26H40O2Si2	57-63-6	2847
1H-Benzotriazole, TMS derivative	C8H10N3OSi	43183-36-4	1725
2,3,4,6-Tetrachlorophenol, TMS derivative	C9H10Cl4OSi	58-90-2	1695
2,3,4-Trichlorophenol, TMS derivative	C9H11Cl3OSi	15950-66-0	1584
2,3,5,6-Tetrachlorophenol, TMS derivative	C9H10Cl4OSi	935-95-5	1684
2,3,5-Trichlorophenol, TMS derivative	C9H11Cl3OSi	933-78-8	1519
2,3,6-Trichlorophenol, TMS derivative	C9H11Cl3OSi	933-75-5	1535
2,3-Dichlorophenol, TMS derivative	C9H12Cl2OSi	576-24-9	1402
2,3-Methylenedioxyamphetamine (MDA), TMS derivative	C13H21NO2Si	23693-17-6	1647
2,4,5-TP (Fenoprop), TMS derivative	C12H15Cl3O3Si	93-72-1	1848
2,4,5-Trichloroaniline, TMS derivative	C9H12Cl3NSi	636-30-6	1645
2,4,6-Trichlorophenol, TMS derivative	C9H11Cl3OSi	88-06-2	1498
2,4-Dichlorophenoxyacetic acid (2,4-D), TMS derivative	C11H14Cl2O3Si	94-75-7	1757
2,4-Dichlorophenylacetic acid, TMS derivative	C11H13Cl2O3Si	19719-28-9	1622
2,5-Dichlorophenol, TMS derivative	C9H12Cl2OSi	583-78-8	1356
2,6-Dibromophenol, TMS derivative	C9H12Br2OSi	608-33-3	1539
2,6-Dichlorophenol, TMS derivative	C9H12Cl2OSi	87-65-0	1354
2,6-Di-tert-butyl-4-hydroxy-4-methyl-2,5-cyclohexadien-1-one (BHT-OH), TMS derivative	C18H32O2Si	10396-80-2	1799
2-Aminobenzothiazole, TMS derivative	C10H14N2SSi	136-95-8	995
2-Chlorophenol, TMS derivative	C9H13ClOSi	95-57-8	1207
2-Ethylhexyl salicylate, TMS derivative	C18H31O3Si	118-60-5	1958
2-Hydroxy-4-metoxybenzophenone, TMS derivative	C17H20O3Si	131-57-7	2107
2-Mercaptobenzothiazol, TMS derivative	C10H15NS2Si	149-30-4	927
2-tert-Butyl-1,4-dihydroxybenzene (TBHQ), 2TMS derivative	C16H30O2Si2	1948-33-0	1620
2-tert-Butyl-1,4-dihydroxybenzene (TBHQ), TMS derivative	C13H22O2Si	1948-33-0	1550
3,4-Dichlorophenol, TMS derivative	C9H12Cl2OSi	95-77-2	1412
3,4-Methylenedioxyamphetamine (MDMA), TMS derivative	C14H23NO2Si	42542-10-9	1725
3,4-Methylenedioxy-N-ethylamphetamine (MDEA), TMS derivative	C15H25NO2Si	82801-81-8	1821
3,5-Dichlorophenol, TMS derivative	C9H12Cl2OSi	591-35-5	1375
3,5-Di-tert-butyl-4-hydroxybenzaldehyde (BHT-CHO), TMS derivative	C18H30O2Si	1620-98-0	1982
3,5-Di-tert-butyl-4-hydroxybenzoic acid (BHT-COOH), TMS derivative	C18H30O3Si	1421-49-4	1941
4-(2,4-dichlorophenoxy)butyric acid (2,4-DB), TMS derivative	C13H18Cl2O3Si	94-82-6	1981

Name	Formula	CAS No.*	Retention Index
4-(4-Chloro-2-methylphenoxy)butanoic acid (MCPB), TMS derivative	C14H21ClO3Si	94-81-5	1916
4-Chloro-2-methylaniline, TMS derivative	C10H16ClNSi	95-69-2	1474
4-Chloroaniline, TMS derivative	C9H14ClNSi	106-47-8	1416
4-Chlorophenol, TMS derivative	C9H13ClOSi	106-48-9	1242
4-Hydroxybenzoic acid propyl ester, TMS derivative	C13H20O3Si	94-13-3	1603
4-Hydroxybenzoic acid, 2TMS derivative	C13H22O3Si2	99-96-7	1638
4-Nonylphenol, TMS derivative	C18H32OSi	104-40-5	1928
4-Octylphenol, TMS derivative	C17H30OSi	1806-26-4	1827
Aminoantipyrine, 2TMS derivative	C17H29N3OSi2	83-07-8	2173
Amphetamine, TMS derivative	C12H21NSi	300-62-9	1309
Atenolol, 2TMS derivative	C20H38N2O3Si2	29122-68-7	2439
Benzoylecgonine, TMS derivative	C19H27NO4Si	519-09-5	2323
Benzyl 4-hydroxybenzoate, TMS derivative	C17H20O3Si	94-18-8	2189
Benzyl salicylate, TMS derivative	C17H20O3Si	118-58-1	2046
Bis(1,3-dichloro-2-propyl) phosphate (BDCPP), TMS derivative	C9H18Cl4O4PSi	72236-72-7	1960
Bis(1-chloro-2-propyl) phosphate (BCPP), TMS derivative	C9H20Cl2O4PSi	789440-10-4	1618
Bis(2-chloroethyl) phosphate (BCEP), TMS derivative	C7H17Cl2O4PSi	3040-56-0	1591
Bisphenol A, 2TMS derivative	C21H32O2Si2	80-05-7	2236
Bisphenol AF, 2TMS derivative	C21H26F6O2Si2	155435-66-8	2030
Bisphenol B, 2TMS derivative	C22H34O2Si2	77-40-7	2317
Bisphenol E, 2TMS derivative	C20H30O2Si2	66-32-8	2200
Bisphenol F, 2TMS derivative	C19H28O2Si2	620-92-8	2170
Bromazepam, TMS derivative	C17H18BrN3OSi	1812-30-2	2503
Butyl 4-hydroxybenzoate, TMS derivative	C14H22O3Si	94-26-8	1776
Butylated hydroxyanisole (BHA), TMS derivative	C14H28O2Si	121-00-6	1552
Carbamazepine, TMS derivative	C18H20N2OSi	298-46-4	2300
Codeine, TMS derivative	C21H29NO3Si	76-57-3	2524
Dibenzyl phosphate, TMS derivative	C17H23O4PSi	1623-08-1	2056
Dibutyl phosphate, TMS derivative	C8H66O4PSi	107-66-4	1520
Diclofenac, TMS derivative	C17H19Cl2NO2Si	15307-86-5	2327
Diethylstilbestrol, 2TMS derivative	C24H36O2Si2	22610-99-7	2387
Diphenyl phosphate, TMS derivative	C15H19O4PSi	838-85-7	2004
Ecgonine methyl ester, TMS derivative	C13H25NO3Si	1915-21-1	1594
Estriol, 3TMS derivative	C27H48O3Si3	50-27-1	2945
Estrone, TMS derivative	C21H30O2Si	53-16-7	2682
Ethyl 4-hydroxybenzoate, TMS derivative	C12H18O3Si	120-47-8	1574
Fenoprofen, TMS derivative	C18H22O3Si	29679-58-1	2003
Fluoxetine, TMS derivative	C20H26F3NOSi	54910-89-3	2036
Ibuprofen, TMS derivative	C16H26O2Si	15687-27-1	1632
Indomethacin, TMS derivative	C22H24ClNO4Si	53-86-1	2984
Isobutyl 4-hydroxybenzoate, TMS derivative	C14H22O3Si	4247-02-3	1729
Ketamine, TMS derivative	C16H24ClNOSi	6740-88-1	1807
Ketoprofen, TMS derivative	C19H22O3Si	22071-15-4	1103

Name	Formula	CAS No.*	Retention Index
Lorazepam, 2TMS derivative	C21H26Cl2N2O2Si2	846-49-1	2553
Lormetazepam, TMS derivative	C19H20Cl2N2O2Si	848-75-9	2764
Meclofenamic acid, TMS derivative	C17H19Cl2NO2Si	644-62-2	2416
Mephedrone, TMS derivative	C14H23NOSi	1189805-46-6	1649
Mestranol, TMS derivative	C24H34O2Si	72-33-3	2785
Methyl 3,5-dibromo-4-hydroxybenzoate, TMS derivative	C11H14Br2O3Si	41727-47-3	1958
Methyl 3-chloro-4-hydroxybenzoate, TMS derivative	C11H15ClO3Si	3964-57-6	1637
Methyl 4-hydroxybenzoate, TMS derivative	C11H16O3Si	99-76-3	1502
Methylphenidate, TMS derivative	C17H27NO2Si	113-45-1	1911
Monobenzyl phthalate, TMS derivative	C18H20O4Si	2528-16-7	2226
Monobutyl phthalate, TMS derivative	C15H22O4Si	131-70-4	1836
Monoisobutyl phthalate, TMS derivative	C15H22O4Si	30833-53-5	1787
Monomethyl phthalate, TMS derivative	C12H16O4Si	4376-18-5	1594
Morphine, 2TMS derivative	C23H35NO3Si2	57-27-2	2579
Naproxen, TMS derivative	C17H22O3Si	22204-53-1	2099
N-Desmethylcitalopram, TMS derivative	C22H27FN2OSi	62498-67-3	2629
N-Desmethylmirtazapine, TMS derivative	C16H17N3	61337-68-6	2459
Nordazepam, TMS derivative	C18H19ClN2OSi	1088-11-5	2335
Norfluoxetine, TMS derivative	C19H24F3NOSi	56161-73-0	1994
Norsertraline, TMS derivative	C19H23Cl2NSi	87857-41-8	2607
Oxazepam, 2TMS derivative	C21H27ClN2O2Si2	604-75-1	2437
Paraxanthine, TMS derivative	C10H16N4O2Si	611-59-6	962
Paroxetine, TMS derivative	C22H28FNO3Si	61869-08-7	2702
Pentachlorophenol, TMS derivative	C9H9Cl5OSi	87-86-5	1888
Phenylphosphonic acid, TMS derivative	C12H23O3PSi2	1571-33-1	1606
Propanolol, TMS derivative	C22H24ClNO4Si	525-66-6	2202
Ritalinic acid, 2TMS derivative	C19H33NO2Si2	19395-41-6	1967
Salbutamol, 3TMS derivative	C22H45NO3Si3	18559-94-9	2037
Salicylic acid, 2TMS derivative	C13H22O3Si2	69-72-7	1523
Sertraline, TMS derivative	C20H25Cl2NSi	79617-96-2	2600
ß-Estradiol, 2TMS derivative	C24H40O2Si2	50-28-2	2728
Tetrahydrocannabinol (THC), TMS derivative	C24H38O2Si	26-51-4	2406
Theobromine, TMS derivative	C10H16N4O2Si	83-67-0	959
Theophylline, TMS derivative	C10H16N4O2Si	58-55-9	1925
Tolfenamic acid, TMS derivative	C17H20ClNO2Si	13710-19-5	2296
Trans-3-Hydroxycotinine, TMS derivative	C13H20N2O2Si	34834-67-8	1908
Trenbolone, TMS derivative	C21H30O2Si	10161-33-8	2783
Triclosan, TMS derivative	C15H15Cl3O2Si	3380-34-5	2154
Venlafaxine, TMS derivative	C20H35NO2Si	93413-69-5	2137

*CAS number of the non-derivatized compound

Table S4: List of chemicals included in the modified commercial Agilent RTL Pesticide Library.

Name	Formula	CAS No.	Retention Index
(1R)-cis-Permethrin	C21H20Cl2O3	54774-46-8	2710
(1R)-trans-Permethrin	C21H20Cl2O3	61949-77-7	2729
γ-BHC (Lindane)	C6H6Cl6	58-89-9	1771
γ-Cyhalothrin	C23H19ClF3NO3	76703-62-3	2619
λ-Cyhalothrin	C23H19ClF3NO3	91465-08-6	2619
1,2,3,5-tetrachlorobenzene	C6H2Cl4	634-90-2	1343
1,2,3-Trichlorobenzene	C6H3Cl3	87-61-6	1230
1,2,4,5-tetrachlorobenzene	C6H2Cl4	95-94-3	1344
1,2,4-Trichlorobenzene	C6H3Cl3	120-82-1	1197
1,2-Dibromo-3-chloropropane	C3H5Br2Cl	96-12-8	1102
1,2-Dichlorobenzene (O-Dichlorobenzene)	C6H4Cl2	95-50-1	1052
1,3-Dichlorobenzene (M-Dichlorobenzene)	C6H4Cl2	541-73-1	1022
1,4-Dichlorobenzene (P-Dichlorobenzene)	C6H4Cl2	106-46-7	1028
1-Naphthaleneacetic acid	C12H10O2	86-87-3	1787
1-Naphthol	C10H8O	90-15-3	1515
2,3,4,5-Tetrachloroanisole	C7H4Cl4O	938-86-3	1670
2,3,5,6-Tetrachloroaniline	C6H3Cl4N	3481-20-7	1632
2,3,5-Trimethacarb (Landrin)	C11H15NO2	2655-15-4	1666
2,4,5-T Methyl ester	C9H7Cl3O3	1928-37-6	1793
2,4,5-TP / Fenoprop	C9H7Cl3O3	93-72-1	1888
2,4,6-TCP / 2,4,6-Trichlorophenol	C6H3Cl3O	88-06-2	1362
2,4,6-Tribromoanisole	C7H5Br3O	607-99-8	1626
2,4,6-Tribromophenol	C6H3Br3O	118-79-6	1645
2,4-D Butyl ester	C12H14Cl2O3	94-80-4	1891
2,4-D Ethyl ester	C10H10Cl2O3	533-23-3	1705
2,4-D Methyl ester	C9H8Cl2O3	1928-38-7	1643
2,4-Dimethylphenol (2,4-Xylenol)	C8H10O	105-67-9	1150
2,6-Diisopropylnaphthalene	C16H20	24157-81-1	1734
2-Chlorophenol	C6H5ClO	95-57-8	1005
2-Ethylhexyl diphenylphosphate (Octicizer)	C20H27O4P	1241-94-7	2438
2-Methylphenol	C7H8O	95-48-7	1056
2-Phenylphenol	C12H10O	90-43-7	1524
3,4,5-Trimethacarb	C11H15NO2	2686-99-9	1741
3,4-DCA / 3,4-Dichloroaniline	C6H5Cl2N	95-76-1	1438
3,5-DCA / 3,5-Dichloroaniline	C6H5Cl2N	626-43-7	1411
3-Hydroxycarbofuran	C12H15NO4	16655-82-6	1904
3-Trifluormethylaniline	C7H6F3N	98-16-8	1056
4,4'-Dibromobenzophenone	C13H8Br2O	3988-03-2	2220
4,4'-Dichlorobenzophenone	C13H8Cl2O	90-98-2	2000
4-Methylphenol	C7H8O	106-44-5	1075
4-Nitrophenol	C6H5NO3	100-02-7	1532
8-Hydroxyquinoline	C9H7NO	148-24-3	1370
a-BHC	C6H6Cl6	319-84-6	1708
Acenaphthene	C12H10	83-32-9	1499

Name	Formula	CAS No.	Retention Index
Acenaphthylene	C12H8	208-96-8	1463
Acephate	C4H10NO3PS	30560-19-1	1439
Acequinocyl (AKD-2033)	C24H32O4	57960-19-7	2879
Acetamiprid	C10H11ClN4	135410-20-7	2463
Acetochlor	C14H20ClNO2	34256-82-1	1897
Acibenzolar-S-methyl (BTH)	C8H6N2OS2	135158-54-2	1905
Acifluorfen-methyl	C15H9ClF3NO5	50594-67-7	2252
Aclonifen	C12H9ClN2O3	74070-46-5	2279
Acrinathrin (Rufast)	C26H21F6NO5	103833-18-7	2649
a-Endosulfan	C9H6Cl6O3S	959-98-8	2134
Akton	C12H14Cl3O3PS	1757-18-2	2134
Alachlor	C14H20ClNO2	15972-60-8	1916
Aldrin	C12H8Cl6	309-00-2	1978
Allethrin	C19H26O3	584-79-2	2089
Allidochlor	C8H12ClNO	93-71-0	1298
Ametoctradin	C15H25N5	865318-97-4	2657
Ametryne (Ametrex)	C9H17N5S	834-12-8	1918
Amidithion	C7H16NO4PS2	919-76-6	1972
Aminocarb	C11H16N2O2	2032-59-9	1776
Amisulbrom	C13H13BrFN5O4S2	348635-87-0	2786
Amitraz	C19H23N3	33089-61-1	2599
a-Naphthylacetamide	C12H11NO	86-86-2	1938
Ancymidol (Ancimidol)	C15H16N2O2	12771-68-5	2242
Anilazine	C9H5Cl3N4	101-05-3	2065
Anilofos	C13H19ClNO3PS2	64249-01-0	2523
Anthracene	C14H10	120-12-7	1798
Anthraquinone	C14H8O2	84-65-1	1977
Aramite(I)	C15H23ClO4S	140-57-8	2214
Aramite(II)	C15H23ClO4S	140-57-8	2236
Aspon (NDP)	C12H28O5P2S2	3244-90-4	1995
Athidathion	C8H15N2O4PS3	19691-80-6	2204
Atraton	C9H17N5O	1610-17-9	1737
Atrazine	C8H14ClN5	1912-24-9	1758
Atrazine-desethyl	C6H10ClN5	6190-65-4	1667
Atrazine-desisopropyl (Deisopropylatrazine)	C5H8ClN5	1007-28-9	1651
Azaconazole	C12H11Cl2N3O2	60207-31-0	2216
Azamethiphos	C9H10ClN2O5PS	35575-96-3	2326
Azidithion (Menazon)	C6H12N5O2PS2	78-57-9	2469
Azinphos-ethyl (Guthion ethyl)	C12H16N3O3PS2	2642-71-9	2644
Azinphos-methyl (Guthion)	C10H12N3O3PS2	86-50-0	2559
Aziprotryne	C7H11N7S	4658-28-0	1819
Azobenzene (Bumadizone artifact)	C12H10N2	103-33-3	1632
Azoxystrobin	C22H17N3O5	131860-33-8	3119
BAM / Dichlorbenzamide	C7H5Cl2NO	2008-58-4	1675
Barban	C11H9Cl2NO2	101-27-9	2188

Name	Formula	CAS No.	Retention Index
BBP / Benzyl butyl phthalate (Butylbenzylphthalate)	C19H20O4	85-68-7	2356
BDMC / 4-Bromo-3,5-dimethylphenyl methylcarbamate	C10H12BrNO2	672-99-1	1866
Beflubutamid	C18H17F4NO2	113614-08-7	2092
Benalaxyl	C20H23NO3	71626-11-4	2342
Benazolin-ethyl	C11H10ClNO3S	25059-80-7	2065
Bendiocarb	C11H13NO4	22781-23-3	1683
Benfluralin	C13H16F3N3O4	1861-40-1	1693
Benfuracarb	C20H30N2O5S	82560-54-1	2655
Benfuresate	C12H16O4S	68505-69-1	1875
Benodanil	C13H10INO	15310-01-7	2296
Benoxacor	C11H11Cl2NO2	98730-04-2	1855
Bensulide	C14H24NO4PS3	741-58-2	1914
Bentazone	C10H12N2O3S	25057-89-0	2018
Benthiavalicarb-isopropyl	C18H24FN3O3S	177406-68-7	2581
Benz[a]anthracene	C18H12	56-55-3	2458
Benzo[a]pyrene	C20H12	50-32-8	2868
Benzo[b]fluoranthene	C20H12	205-99-2	2778
Benzo[ghi]perylene	C22H12	191-24-2	3256
Benzo[k]fluoranthene	C20H12	207-08-9	2785
Benzoylprop-ethyl	C18H17Cl2NO3	22212-55-1	2452
Benzyladenine (6-Benzylaminopurine)	C12H11N5	1214-39-7	2494
Benzylbenzoate	C14H12O2	120-51-4	1773
Bifenazate (D 2341)	C17H20N2O3	149877-41-8	2490
Bifenox	C14H9Cl2NO5	42576-02-3	2518
Bifenthrin	C23H22ClF3O2	82657-04-3	2490
Binapacryl	C15H18N2O6	485-31-4	2246
Bioallethrin (Esbiothrin)	C19H26O3	28434-00-6	2087
Bioresmethrin (Isatrin)	C22H26O3	28434-01-7	2423
Biphenyl	C12H10	92-52-4	1392
Bis(2,3,3,3-tetrachloropropyl)ether	C6H6Cl8O	127-90-2	1929
Bitertanol	C20H23N3O2	55179-31-2	2696
Bixafen	C18H12Cl2F3N3O	581809-46-3	2902
Boscalid (Nicobifen)	C18H12Cl2N2O	188425-85-6	2837
Bromacil	C9H13BrN2O2	314-40-9	1963
Bromethalin	C14H7Br3F3N3O4	63333-35-7	2912
Bromfenvinphos (E)	C12H14BrCl2O4P	33399-00-7	2168
Bromfenvinphos (Z)	C12H14BrCl2O4P	58580-13-5	2168
Bromfenvinphos-methyl	C10H10BrCl2O4P	13104-21-7	2081
Bromobutide	C15H22BrNO	74712-19-9	1887
Bromociclen	C8H5BrCl6	1715-40-8	1846
Bromophos	C8H8BrCl2O3PS	2104-96-3	2032
Bromophos-ethyl	C10H12BrCl2O3PS	4824-78-6	2123
Bromopropylate	C17H16Br2O3	18181-80-1	2475
Bromoxynil	C7H3Br2NO	1689-84-5	1682
Bromoxynil octanoate	C15H17Br2NO2	1689-99-2	2347

Name	Formula	CAS No.	Retention Index
Bromuconazole(I)	C13H12BrCl2N3O	116255-48-2	2462
Bromuconazole(II)	C13H12BrCl2N3O	116255-48-2	2520
Bufencarb	C13H19NO2	8065-36-9	1776
Bupirimate	C13H24N4O3S	41483-43-6	2224
Buprofezin	C16H23N3OS	69327-76-0	2214
Butachlor (Machete)	C17H26ClNO2	23184-66-9	2149
Butafenacil	C20H18ClF3N2O6	134605-64-4	2772
Butamifos	C13H21N2O4PS	36335-67-8	2166
Butralin (Sutralin)	C14H21N3O4	33629-47-9	2034
Buturon	C12H13ClN2O	3766-60-7	2015
Butylate (Sutan)	C11H23NOS	2008-41-5	1436
Cadusafos	C10H23O2PS2	95465-99-9	1695
Cafenstrole	C16H22N4O3S	125306-83-4	2786
Captafol	C10H9Cl4NO2S	2425-06-1	2403
Captan	C9H8Cl3NO2S	133-06-2	2078
Carbaryl	C12H11NO2	63-25-2	1908
Carbetamide	C12H16N2O3	16118-49-3	2008
Carbofuran	C12H15NO3	1563-66-2	1752
Carbofuran phenol (Carbofuranphenol)	C10H12O2	1563-38-8	1310
Carbofuran-3-keto	C12H13NO4	16709-30-1	1841
Carbophenothion	C11H16ClO2PS3	786-19-6	2337
Carbosulfan	C20H32N2O3S	55285-14-8	2477
Carboxin	C12H13NO2S	5234-68-4	2204
Carfentrazone-ethyl	C15H14Cl2F3N3O3	128639-02-1	2349
Carpropamide (KTU 3616)	C15H18Cl3NO	104030-54-8	2248
Carvone	C10H14O	99-49-0	1255
CDEC / Sulfallate	C8H14ClNS2	95-06-7	1708
Chlomethoxyfen	C13H9Cl2NO4	32861-85-1	2454
Chloramben-methyl	C8H7Cl2NO2	7286-84-2	1770
Chlorantraniliprole	C18H14BrCl2N5O2	500008-45-7	2491
Chlorbenside	C13H10Cl2S	103-17-3	2099
Chlorbenside sulfone	C13H10Cl2O2S	7082-99-7	2324
Chlorbicyclen	C9H6Cl8	2550-75-6	2066
Chlorbromuron	C9H10BrClN2O2	13360-45-7	2066
Chlorbufam	C11H10ClNO2	1967-16-4	1754
Chlordane-cis (a-Chlordan)	C10H6Cl8	5103-71-9	2141
Chlordane-trans (?-Chlordan)	C10H6Cl8	5103-74-2	2109
Chlordecone	C10Cl10O	143-50-0	2305
Chlordene	C10H6Cl6	3734-48-3	1804
Chlordimeform	C10H13ClN2	6164-98-3	1665
Chlorethoxyfos	C6H11Cl4O3PS	54593-83-8	1631
Chlorfenapyr	C15H11BrClF3N2O	122453-73-0	2251
Chlorfenethol	C14H12Cl2O	80-06-8	2069
Chlorfenson (Ovex)	C12H8Cl2O3S	80-33-1	2158
Chlorfenvinphos(I)	C12H14Cl3O4P	470-90-6	2084

Name	Formula	CAS No.	Retention Index
Chlorflurazurone (Chlorfluazurone)	C20H9Cl3F5N3O3	71422-67-8	1966
Chlorflurenol-methyl	C15H11ClO3	2536-31-4	2107
Chloridazon (PAC)	C10H8ClN3O	1698-60-8	2360
Chlorimuron-ethyl	C15H15ClN4O6S	90982-32-4	1881
Chlormephos	C5H12ClO2PS2	24934-91-6	1447
Chlornitrofen	C12H6Cl3NO3	1836-77-7	2329
Chlorobenzilate	C16H14Cl2O3	510-15-6	2260
Chloroneb	C8H8Cl2O2	2675-77-6	1518
Chloropropham (Chlorpropham)	C10H12ClNO2	101-21-3	1658
Chloropropylate	C17H16Cl2O3	5836-10-2	2262
Chlorothalonil	C8Cl4N2	1897-45-6	1828
Chloroxuron	C15H15ClN2O2	1982-47-4	1925
Chlorphenprop-methyl (Fatex)	C10H10Cl2O2	14437-17-3	1599
Chlorpyrifos	C9H11Cl3NO3PS	2921-88-2	1999
Chlorpyrifos oxon	C9H11Cl3NO4P	5598-15-2	1988
Chlorpyrifos-methyl	C7H7Cl3NO3PS	5598-13-0	1900
Chlorthiamid	C7H5Cl2NS	1918-13-4	1879
Chlorthion	C8H9ClNO5PS	500-28-7	2019
Chlorthiophos	C11H15Cl2O3PS2	21923-23-9	2301
Chlortoluron (Chlorotoluron)	C10H13ClN2O	15545-48-9	1968
Chrysene	C18H12	218-01-9	2468
Cinidon-ethyl	C19H17Cl2NO4	142891-20-1	3229
Cinmethylin	C18H26O2	87818-31-3	1925
Ciodrin (Crotoxyphos)	C14H19O6P	7700-17-6	2105
cis-1,2,3,6-Tetrahydrophthalimide	C8H9NO2	1469-48-3	1487
Climbazole	C15H17ClN2O2	38083-17-9	2146
Clodinafop-propargyl	C17H13ClFNO4	105512-06-9	2370
Clomazone	C12H14ClNO2	81777-89-1	1761
Clomeprop	C16H15Cl2NO2	84496-56-0	2526
Cloquintocet-mexyl	C18H22ClNO3	99607-70-2	2491
Coumafuryl	C17H14O5	117-52-2	2474
Coumaphos	C14H16ClO5PS	56-72-4	2738
Coumatetralyl	C19H16O3	5836-29-3	2704
Crimidine	C7H10ClN3	535-89-7	1526
Crufomate (Ruelene)	C12H19ClNO3P	299-86-5	2023
Cyanazine (Fortrol)	C9H13ClN6	21725-46-2	2001
Cyanofenphos (Cyanophenphos)	C15H14NO2PS	13067-93-1	2347
Cyanophos	C9H10NO3PS	2636-26-2	1785
Cycloate	C11H21NOS	1134-23-2	1642
Cycloxydim (Focus)	C17H27NO3S	101205-02-1	2678
Cycluron	C11H22N2O	2163-69-1	1779
Cyenopyrafen	C24H31N3O2	560121-52-0	2532
Cyflee (Cythioate)	C8H12NO5PS2	115-93-5	2391
Cyflufenamid	C20H17F5N2O2	180409-60-3	2247
Cyfluthrin(I)	C22H18Cl2FNO3	68359-37-5	2794

Name	Formula	CAS No.	Retention Index
Cyfluthrin(II)	C22H18Cl2FNO3	68359-37-5	2808
Cyfluthrin(III)	C22H18Cl2FNO3	68359-37-5	2818
Cyfluthrin(IV)	C22H18Cl2FNO3	68359-37-5	2824
Cyhalofop-butyl	C20H20FNO4	122008-85-9	2589
Cypermethrin(I)	C22H19Cl2NO3	52315-07-8	2837
Cypermethrin(II)	C22H19Cl2NO3	52315-07-8	2851
Cypermethrin(III)	C22H19Cl2NO3	52315-07-8	2862
Cypermethrin(IV)	C22H19Cl2NO3	52315-07-8	2867
Cyphenothrin	C24H25NO3	39515-40-7	2676
Cyprazine	C9H14ClN5	22936-86-3	1883
Cyproconazole(I)	C15H18ClN3O	94361-06-5	2236
Cyprodinil	C14H15N3	121552-61-2	2049
Cyprofuram	C14H14ClNO3	69581-33-5	2274
Cyromazine	C6H10N6	66215-27-8	1763
Daimuron (Dymron)	C17H20N2O	42609-52-9	2374
Dazomet	C5H10N2S2	533-74-4	1727
DBA / Dibenz[a,h]anthracene	C22H14	53-70-3	3202
d-BHC	C6H6Cl6	319-86-8	1818
DBP / Dibutyl phthalate	C16H22O4	84-74-2	1967
DCPA / Chlorthal-dimethyl	C10H6Cl4O4	1861-32-1	2008
DDM / Dichlorophen	C13H10Cl2O2	97-23-4	2459
DEET / Diethyltoluamide	C12H17NO	134-62-3	1586
Deltamethrin (Decamethrin)	C22H19Br2NO3	52918-63-5	3076
Demeton-O	C8H19O3PS2	298-03-3	1622
Demeton-S	C8H19O3PS2	126-75-0	1734
Demeton-S-methyl	C6H15O3PS2	919-86-8	1628
Demeton-S-methylsulfone	C6H15O5PS2	17040-19-6	1938
Desmedipham	C16H16N2O4	13684-56-5	1723
Desmetryn	C8H15N5S	1014-69-3	1875
Desthio-Prothioconazole(I)	C14H15Cl2N3O	120983-64-4	2217
Diafenthiuron	C23H32N2OS	80060-09-9	2270
Dialifos	C14H17ClNO4PS2	10311-84-9	2660
Diallate (cis)	C10H17Cl2NOS	2303-16-4	1702
Diallate (trans)	C10H17Cl2NOS	17708-58-6	1717
Diazinon (Dimpylate)	C12H21N2O3PS	333-41-5	1813
Diazoxon	C12H21N2O4P	962-58-3	1787
Dibutyl succinate	C12H22O4	141-03-7	1560
Dicapthon	C8H9ClNO5PS	2463-84-5	2007
Dichlobenil	C7H3Cl2N	1194-65-6	1357
Dichlobutrazol (Diclobutrazol)	C15H19Cl2N3O	75736-33-3	2211
Dichlofenthion	C10H13Cl2O3PS	97-17-6	1883
Dichlofluanid	C9H11Cl2FN2O2S2	1085-98-9	1970
Dichlofluanid metabolite (DMSA)	C8H12N2O2S	4710-17-2	1662
Dichlone	C10H4Cl2O2	117-80-6	1807
Dichloran (Dicloran)	C6H4Cl2N2O2	99-30-9	1730

Name	Formula	CAS No.	Retention Index
Dichlormid	C8H11Cl2NO	37764-25-3	1364
Dichlorprop-methyl	C10H10Cl2O3	57153-17-0	1622
Dichlorvos	C4H7Cl2O4P	62-73-7	1254
Diclocymet(I)	C15H18Cl2N2O	139920-32-4	2086
Diclocymet(II)	C15H18Cl2N2O	139920-32-4	2122
Diclofop-methyl	C16H14Cl2O4	51338-27-3	2400
Dicofol	C14H9Cl5O	115-32-2	2483
Dicrotophos (Bidrin)	C8H16NO5P	141-66-2	1680
Dieldrin	C12H8Cl6O	60-57-1	2188
Diethatyl-ethyl (Antor)	C16H22ClNO3	38727-55-8	2166
Diethofencarb	C14H21NO4	87130-20-9	1991
Difenoconazole(I)	C19H17Cl2N3O3	119446-68-3	3024
Difenoconazole(II)	C19H17Cl2N3O3	119446-68-3	3034
Difenoxuron	C16H18N2O3	14214-32-5	1986
Diflovidazin (SZ1-121)	C14H7ClF2N4	162320-67-4	2318
Diflufenican	C19H11F5N2O2	83164-33-4	2408
Dimefox	C4H12FN2OP	115-26-4	1039
Dimefuron	C15H19ClN4O3	34205-21-5	2051
Dimepiperate	C15H21NOS	61432-55-1	2086
Dimethachlor	C13H18ClNO2	50563-36-5	1883
Dimethametryn	C11H21N5S	22936-75-0	2066
Dimethenamid (SAN 582H)	C12H18ClNO2S	87674-68-8	1885
Dimethenamid-P	C12H18ClNO2S	163515-14-8	1885
Dimethipin	C6H10O4S2	55290-64-7	1756
Dimethoate	C5H12NO3PS2	60-51-5	1736
Dimethomorph (E)	C21H22ClNO4	110488-70-5	3122
Dimethylvinphos	C10H10Cl3O4P	2274-67-1	1996
Dimetilan	C10H16N4O3	644-64-4	1919
Dimoxystrobin	C19H22N2O3	149961-52-4	2480
Diniconazole(I)	C15H17Cl2N3O	83657-24-3	2272
Dinitramine	C11H13F3N4O4	29091-05-2	1827
Dinobuton	C14H18N2O7	973-21-7	2098
Dinocap(I)	C18H24N2O6	39300-45-3	2445
Dinocap(II)	C18H24N2O6	39300-45-3	2473
Dinocap(III)	C18H24N2O6	39300-45-3	2500
Dinoseb (Subitex)	C10H12N2O5	88-85-7	1817
Dinoseb acetate	C12H14N2O6	2813-95-8	1934
Dinoterb	C10H12N2O5	1420-07-1	1798
Dinoterb acetate	C12H14N2O6	3204-27-1	1949
Diofenolan(I)	C18H20O4	63837-33-2	2344
Diofenolan(II)	C18H20O4	63837-33-2	2359
Dioxabenzofos	C8H9O3PS	3811-49-2	1677
Dioxacarb	C11H13NO4	6988-21-2	1875
Dioxathion	C12H26O6P2S4	78-34-2	2754
Diphenadione (Diphacinone)	C23H16O3	82-66-6	2964

Name	Formula	CAS No.	Retention Index
Diphenamid	C16H17NO	957-51-7	2035
Dipropetryn	C11H21N5S	4147-51-7	1978
Dipropyl isocinchomeronate (MGK-326)	C13H17NO4	136-45-8	1901
Disugran (Dicamba-methyl)	C9H8Cl2O3	6597-78-0	1528
Disulfoton	C8H19O2PS3	298-04-4	1818
Disulfoton sulfone	C8H19O4PS3	2497-06-5	2139
Ditalimfos (Plondrel)	C12H14NO4PS	5131-24-8	2151
Dithiopyr	C15H16F5NO2S2	97886-45-8	1954
Diuron	C9H10Cl2N2O	330-54-1	2079
DiuronMetabolite[3,4-Dichlorophenylisocyanate]	C7H3Cl2NO	102-36-3	1318
DMDE / Methoxychlor olefin (Methoxychlor (-HCl))	C16H14Cl2O2	2132-70-9	2344
DMDT / Metox (Methoxychlor)	C16H15Cl3O2	72-43-5	2493
DMST / N,N-Dimethyl-N'-p-tolylsulfonyldiamide	C9H14N2O2S	66840-71-9	1768
DNOC / 2,4-Dinitro-o-kresol	C7H6N2O5	534-52-1	1620
Dodemorph	C18H35NO	1593-77-7	2026
DPA / Diphenylamine (DFA)	C12H11N	122-39-4	1628
Drazoxolon	C10H8ClN3O2	5707-69-7	2027
e-BHC	C6H6Cl6	6108-10-7	1837
EDPP / Edifenphos	C14H15O2PS2	17109-49-8	2345
Empenthrin(I)	C18H26O2	54406-48-3	1733
Empenthrin(II)	C18H26O2	54406-48-3	1720
Endosulfan ether	C9H6Cl6O	3369-52-6	1851
Endosulfan sulfate	C9H6Cl6O4S	1031-07-8	2348
Endothal	C8H10O5	145-73-3	1500
Endrin	C12H8Cl6O	72-20-8	2234
Endrin aldehyde	C12H8Cl6O	7421-93-4	2296
Endrin ketone	C12H8Cl6O	53494-70-5	2450
EPN / Tsumaphos	C14H14NO4PS	2104-64-5	2478
Epoxiconazole (BAS 480F)	C17H13ClFN3O	133855-98-8	2430
EPTC / Epthame	C9H19NOS	759-94-4	1363
Erbon	C11H9Cl5O3	136-25-4	2231
Esfenvalerate	C25H22ClNO3	66230-04-4	2997
Esprocarb	C15H23NOS	85785-20-2	1963
Etaconazole(I)	C14H15Cl2N3O2	60207-93-4	2274
Etaconazole(II)	C14H15Cl2N3O2	60207-93-4	2282
Ethalfluralin	C13H14F3N3O4	55283-68-6	1671
Ethiofencarb	C11H15NO2S	29973-13-5	1860
Ethiolate	C7H15NOS	2941-55-1	1209
Ethion	C9H22O4P2S4	563-12-2	2294
Ethiozin	C9H16N4OS	64529-56-2	1937
Ethofumesate	C13H18O5S	26225-79-6	1964
Ethofumesate-2-keto	C11H12O5S	26244-33-7	1883
Ethoprophos (Ethoprop)	C8H19O2PS2	13194-48-4	1642
Ethoxyquin	C14H19NO	91-53-2	1743
Ethylchlozate	C11H11ClN2O2	27512-72-7	2067

Name	Formula	CAS No.	Retention Index
Etobenzanid	C16H15Cl2NO3	79540-50-4	2767
Etofenprox	C25H28O3	80844-07-1	2877
Etoxazole	C21H23F2NO2	153233-91-1	2507
Etridiazole (Echlomezole)	C5H5Cl3N2OS	2593-15-9	1464
Etrimfos	C10H17N2O4PS	38260-54-7	1841
Eugenol	C10H12O2	97-53-0	1364
Famoxadone	C22H18N2O4	131807-57-3	3119
Famphur (Famophos)	C10H16NO5PS2	52-85-7	2335
Fenamidone	C17H17N3OS	161326-34-7	2509
Fenamiphos	C13H22NO3PS	22224-92-6	2165
Fenamiphos sulfone	C13H22NO5PS	31972-44-8	2462
Fenamiphos sulfoxide	C13H22NO4PS	31972-43-7	2449
Fenarimol	C17H12Cl2N2O	60168-88-9	2623
Fenazaflor	C15H7Cl2F3N2O2	14255-88-0	2310
Fenazaflor metabolite	C8H3Cl2F3N2	2338-25-2	1736
Fenazaquin	C20H22N2O	120928-09-8	2513
Fenbuconazole	C19H17ClN4	114369-43-6	2784
Fenchlorazole-ethyl	C12H8Cl5N3O2	103112-35-2	2523
Fenchlorphos (Ronnel)	C8H8Cl3O3PS	299-84-3	1929
Fenclorim	C10H6Cl2N2	3740-92-9	1708
Fenfluthrin	C15H11Cl2F5O2	75867-00-4	1873
Fenfuram	C12H11NO2	24691-80-3	1820
Fenhexamid	C14H17Cl2NO2	126833-17-8	2355
Fenitrothion	C9H12NO5PS	122-14-5	1957
Fenobucarb (Baycarb)	C12H17NO2	3766-81-2	1617
Fenoprop-methyl	C10H9Cl3O3	4841-20-7	1765
Fenothiocarb	C13H19NO2S	62850-32-2	2129
Fenoxanil	C15H18Cl2N2O2	115852-48-7	2252
Fenoxaprop-ethyl	C18H16ClNO5	66441-23-4	2672
Fenoxaprop-P-ethyl	C18H16ClNO5	71283-80-2	2671
Fenoxycarb	C17H19NO4	79127-80-3	2478
Fenpiclonil	C11H6Cl2N2	74738-17-3	2449
Fenpropathrin	C22H23NO3	39515-41-8	2505
Fenpropidin	C19H31N	67306-00-7	1940
Fenpropimorph	C20H33NO	67564-91-4	1998
Fenpyroximate	C24H27N3O4	134098-61-6	1749
Fenson	C12H9ClO3S	80-38-6	2018
Fensulfothion	C11H17O4PS2	115-90-2	2270
Fensulfothion sulfone	C11H17O5PS2	14255-72-2	2306
Fenthion	C10H15O3PS2	55-38-9	1995
Fenthion oxon sulfone	C10H15O6PS	14086-35-2	2210
Fenthion sulfone	C10H15O5PS2	3761-42-0	2283
Fenthion sulfoxide (Mesulfenfos)	C10H15O4PS2	3761-41-9	2270
Fenuron (N,N-Dimethyl-N-phenylurea)	C9H12N2O	101-42-8	1643
Fenvalerate	C25H22ClNO3	51630-58-1	2967

Name	Formula	CAS No.	Retention Index
Ferimzone	C15H18N4	89269-64-7	2109
Fipronil	C12H4Cl2F6N4OS	120068-37-3	2090
Fipronil sulfide	C12H4Cl2F6N4S	120067-83-6	2075
Fipronil sulfone	C12H4Cl2F6N4O2S	120068-36-2	2221
Flamprop-isopropyl	C19H19ClFNO3	52756-22-6	2282
Flamprop-methyl	C17H15ClFNO3	52756-25-9	2212
Flocoumafen	C33H25F3O4	90035-08-8	2814
Flonicamid	C9H6F3N3O	158062-67-0	1612
Fluacrypyrim	C20H21F3N2O5	229977-93-9	2331
Fluazifop	C15H12F3NO4	69335-91-7	2026
Fluazifop-butyl	C19H20F3NO4	69806-50-4	2251
Fluazifop-methyl	C16H14F3NO4	69335-90-6	2027
Fluazifop-P-butyl	C19H20F3NO4	79241-46-6	2251
Fluazinam (Shirlan)	C13H4Cl2F6N4O4	79622-59-6	2081
Flubenzimine	C17H10F6N4S	37893-02-0	2197
Fluchloralin	C12H13ClF3N3O4	33245-39-5	1820
Flucythrinate(I)	C26H23F2NO4	70124-77-5	2870
Flucythrinate(II)	C26H23F2NO4	70124-77-5	2898
Fludioxonil	C12H6F2N2O2	131341-86-1	2188
Flufenacet	C14H13F4N3O2S	142459-58-3	2011
Flufenoxuron	C21H11ClF6N2O3	101463-69-8	1845
Flumetralin	C16H12ClF4N3O4	62924-70-3	2152
Flumiclorac pentyl	C21H23ClFNO5	87546-18-7	3101
Flumioxazin	C19H15FN2O4	103361-09-7	2970
Fluometuron	C10H11F3N2O	2164-17-2	1645
Fluopicolide	C14H8Cl3F3N2O	239110-15-7	2378
Fluopyram	C16H11ClF6N2O	658066-35-4	2087
Fluoranthene	C16H10	206-44-0	2067
Fluorene	C13H10	86-73-7	1594
Fluoroglycofen-ethyl	C18H13ClF3NO7	77501-90-7	2652
Fluoroimide	C10H4Cl2FNO2	41205-21-4	1733
Fluotrimazole	C22H16F3N3	31251-03-3	2428
Fluoxastrobin	C21H16ClFN4O5	193740-76-0	3315
Fluquinconazole(I)	C16H8Cl2FN5O	136426-54-5	2735
Flurenol-butyl	C18H18O3	2314-09-2	2113
Flurenol-methyl	C15H12O3	1216-44-0	1922
Fluridone	C19H14F3NO	59756-60-4	2924
Flurochloridone	C12H10Cl2F3NO	61213-25-0	2024
Flurodifen (Fluorodifen)	C13H7F3N2O5	15457-05-3	2172
Fluroxypyr 1-methylheptyl ester (Starane)	C15H21Cl2FN2O3	81406-37-3	2404
Flurprimidol	C15H15F3N2O2	56425-91-3	1887
Flurtamone	C18H14F3NO2	96525-23-4	2555
Flusilazol	C16H15F2N3Si	85509-19-9	2216
Flusulfamide	C13H7Cl2F3N2O4S	106917-52-6	2502
Fluthiacet-methyl	C15H15ClFN3O3S2	117337-19-6	3249

Name	Formula	CAS No.	Retention Index
Flutolanil	C17H16F3NO2	66332-96-5	2174
Flutriafol	C16H13F2N3O	76674-21-0	2152
Fluvalinate	C26H22ClF3N2O3	69409-94-5	2998
Fluxapyroxad	C18H12F5N3O	907204-31-3	2488
Folpet	C9H4Cl3NO2S	133-07-3	2091
Fonofos	C10H15OPS2	944-22-9	1790
Formothion	C6H12NO4PS2	2540-82-1	1858
Fosthiazate	C9H18NO3PS2	98886-44-3	2032
Fthalide (Tetrachlorophthalide)	C8H2Cl4O2	27355-22-2	2023
Fuberidazole	C11H8N2O	3878-19-1	1898
Furalaxyl	C17H19NO4	57646-30-7	2097
Furametpyr	C17H20ClN3O2	123572-88-3	2547
Furathiocarb	C18H26N2O5S	65907-30-4	2553
Furilazole	C11H13Cl2NO3	121776-33-8	1747
Furmecyclox	C14H21NO3	60568-05-0	1861
Genite	C12H8Cl2O3S	97-16-5	2145
Halfenprox	C24H23BrF2O3	111872-58-3	2843
Halosulfuron-methyl	C13H15ClN6O7S	100784-20-1	1894
Haloxyfop-ethoxyethyl	C19H19ClF3NO5	87237-48-7	2431
Haloxyfop-methyl	C16H13ClF3NO4	69806-40-2	2129
Haloxyfop-P-methyl	C16H13ClF3NO4	72619-32-0	2129
HCB / Hexachlorobenzene	C6Cl6	118-74-1	1721
Heptachlor	C10H5Cl7	76-44-8	1910
Heptachlor epoxide A (Heptachlor endo-epoxide)	C10H5Cl7O	28044-83-9	2069
Heptachlor epoxide B (Heptachlor exo-epoxide)	C10H5Cl7O	1024-57-3	2060
Heptenophos	C9H12ClO4P	23560-59-0	1586
Hexachlorobutadiene	C4Cl6	87-68-3	1231
Hexaconazole(I)	C14H17Cl2N3O	79983-71-4	2167
Hexaflumuron	C16H8Cl2F6N2O3	86479-06-3	1443
Hexazinone	C12H20N4O2	51235-04-2	2384
Hexythiazox	C17H21ClN2O2S	78587-05-0	2108
Hydroprene	C17H30O2	41096-46-2	1880
Imazalil (Enilconazole)	C14H14Cl2N2O	35554-44-0	2176
Imazamethabenz	C15H18N2O3	100728-84-5	2151
Imazamethabenz-methyl	C16H20N2O3	81405-85-8	2219
Imibenconazole	C17H13Cl3N4S	86598-92-7	3196
Imibenconazole-desbenzyl	C10H8Cl2N4O	154221-27-9	2207
Imiprothrin	C17H22N2O4	72963-72-5	2310
Indanofan	C20H17ClO3	133220-30-1	2511
Indeno[1,2,3-cd]pyrene	C22H12	193-39-5	3187
Indoxacarb	C22H17ClF3N3O7	173584-44-6	3076
Iodofenphos	C8H8Cl2IO3PS	18181-70-9	2169
Ioxynil	C7H3I2NO	1689-83-4	1952
Ioxynil octanoate	C15H17I2NO2	3861-47-0	2622
Ioxynil-methyl	C8H5I2NO	3336-40-1	1899

Name	Formula	CAS No.	Retention Index
IPC / Propham	C10H13NO2	122-42-9	1462
Ipconazole(I)	C18H24ClN3O	125225-28-7	2606
Iprobenfos	C13H21O3PS	26087-47-8	1850
Iprodione (Glycophen)	C13H13Cl2N3O3	36734-19-7	2456
Iprovalicarb(I)	C18H28N2O3	140923-17-7	2206
Iprovalicarb(II)	C18H28N2O3	140923-17-7	2228
Irgarol (Cybutryne)	C11H19N5S	28159-98-0	2083
Isazofos (Miral)	C9H17ClN3O3PS	42509-80-8	1837
Isocarbamide	C8H15N3O2	30979-48-7	1775
Isocarbophos	C11H16NO4PS	24353-61-5	2013
Isodrin	C12H8Cl6	465-73-6	2036
Isofenphos	C15H24NO4PS	25311-71-1	2086
Isofenphos-methyl	C14H22NO4PS	99675-03-3	2048
Isomethiozin	C12H20N4OS	57052-04-7	2002
Isoprocarb(I)	C11H15NO2	2631-40-5	1493
Isoprocarb(II)	C11H15NO2	2631-40-5	1547
Isopropalin	C15H23N3O4	33820-53-0	2051
Isoprothiolane	C12H18O4S2	50512-35-1	2180
Isoproturon	C12H18N2O	34123-59-6	1918
Isopyrazam	C20H23F2N3O	881685-58-1	2670
Isoxaben	C18H24N2O4	82558-50-7	2657
Isoxadifen-ethyl	C18H17NO3	163520-33-0	2330
Isoxaflutole	C15H12F3NO4S	141112-29-0	2172
Isoxathion	C13H16NO4PS	18854-01-8	2235
Jasmone(I)	C11H16O	488-10-8	1398
Jasmone(II)	C11H16O	488-10-8	1407
Kadethrin	C23H24O4S	58769-20-3	3298
Kelevan	C17H12Cl10O4	4234-79-1	2305
Kinoprene	C18H28O2	42588-37-4	1988
Kresoxim-methyl	C18H19NO4	143390-89-0	2228
Lactofen	C19H15ClF3NO7	77501-63-4	2627
Lenacil	C13H18N2O2	2164-08-1	2352
Leptophos	C13H10BrCl2O2PS	21609-90-5	2565
Linuron	C9H10Cl2N2O2	330-55-2	1961
Lufenuron	C17H8Cl2F8N2O3	103055-07-8	1430
Malaoxon	C10H19O7PS	1634-78-2	1908
Malathion	C10H19O6PS2	121-75-5	1981
MCPA-Butotyl (MCPA-butoxyethyl ester)	C15H21ClO4	19480-43-4	2091
MCPA-methyl	C10H11ClO3	2436-73-9	1573
MCPA-thioethyl (Phenothiol)	C11H13ClO2S	25319-90-8	1830
MCPB-methyl	C12H15ClO3	57153-18-1	1792
Mecarbam	C10H20NO5PS2	2595-54-2	2088
Mefenacet (Rancho)	C16H14N2O2S	73250-68-7	2585
Mefenoxam	C15H21NO4	70630-17-0	1928
Mefenpyr-diethyl	C16H18Cl2N2O4	135590-91-9	2446

Name	Formula	CAS No.	Retention Index
Mefluidide	C11H13F3N2O3S	53780-34-0	2056
Mepanipyrim	C14H13N3	110235-47-7	2143
Mephosfolan	C8H16NO3PS2	950-10-7	2080
Mepronil	C17H19NO2	55814-41-0	2310
Merphos	C12H27PS3	150-50-5	2071
Metaflumizone	C24H16F6N4O2	139968-49-3	2258
Metalaxyl	C15H21NO4	57837-19-1	1927
Metamitron	C10H10N4O	41394-05-2	2211
Metazachlor	C14H16ClN3O	67129-08-2	2060
Metconazole(I)	C17H22ClN3O	125116-23-6	2511
Methabenzthiazuron	C10H11N3OS	18691-97-9	1665
Methacrifos	C7H13O5PS	62610-77-9	1512
Methamidophos (Metamidophos)	C2H8NO2PS	10265-92-6	1231
Methfuroxam	C14H15NO2	28730-17-8	2007
Methidathion	C6H11N2O4PS3	950-37-8	2115
Methiocarb (Mercaptodimethur)	C11H15NO2S	2032-65-7	1955
Methiocarb sulfone	C11H15NO4S	2179-25-1	1935
Methiocarb sulfoxide	C11H15NO3S	2635-10-1	2280
Methomyl	C5H10N2O2S	16752-77-5	1573
Methoprene	C19H34O3	40596-69-8	2106
Methoprotryne	C11H21N5OS	841-06-5	2219
Methyl 1-naphthalene acetate	C13H12O2	2876-78-0	1710
Methyl Trithion (Carbophenothion-methyl)	C9H12ClO2PS3	953-17-3	2252
Metobromuron	C9H11BrN2O2	3060-89-7	1857
Metolachlor	C15H22ClNO2	51218-45-2	1988
Metolachlor (S)	C15H22ClNO2	87392-12-9	1988
Metolcarb	C9H11NO2	1129-41-5	1467
Metominostrobin (E)	C16H16N2O3	133408-50-1	2186
Metrafenone	C19H21BrO5	220899-03-6	2668
Metribuzin	C8H14N4OS	21087-64-9	1886
Mevinphos (Phosdrin)	C7H13O6P	7786-34-7	1437
Mexacarbate (Zectran)	C12H18N2O2	315-18-4	1831
MGK 264 (Synergist 264) (Pyrdone)	C17H25NO2	113-48-4	2030
Mirex	C10Cl12	2385-85-5	2576
Molinate	C9H17NOS	2212-67-1	1544
Monalide	C13H18ClNO	7287-36-7	1855
Monocrotophos (Azodrin)	C7H14NO5P	6923-22-4	1692
Monolinuron	C9H11ClN2O2	1746-81-2	1756
Monuron	C9H11ClN2O	150-68-5	1857
Moskene	C14H18N2O4	116-66-5	1896
Musk Ambrette (natural)	C16H28O2	123-69-3	1938
Musk Ketone	C14H18N2O5	81-14-1	1993
Musk Xylol (Musk Xylene)	C12H15N3O6	81-15-2	1873
MuskTebetine	C13H18N2O4	145-39-1	1949
Myclobutanil	C15H17ClN4	88671-89-0	2208

Name	Formula	CAS No.	Retention Index
N-(2,4-Dimethylphenyl)formamide	C9H11NO	60397-77-5	1485
Naled (Dibrom)	C4H7Br2Cl2O4P	300-76-5	1665
Naphthalene	C10H8	91-20-3	1205
Naphthalic anhydride	C12H6O3	81-84-5	2041
Naproanilide	C19H17NO2	52570-16-8	2617
Napropamide	C17H21NO2	15299-99-7	2164
Nicotine	C10H14N2	54-11-5	1358
Nitenpyram	C11H15ClN4O2	150824-47-8	1838
Nitralin (Planavin)	C13H19N3O6S	4726-14-1	2437
Nitrapyrin	C6H3Cl4N	1929-82-4	1461
Nitrofen	C12H7Cl2NO3	1836-75-5	2234
Nitrothal-isopropyl	C14H17NO6	10552-74-6	2019
Nonachlor-cis	C10H5Cl9	5103-73-1	2284
Nonachlor-trans	C10H5Cl9	39765-80-5	2151
Norflurazon	C12H9ClF3N3O	27314-13-2	2355
Norflurazon-desmethyl	C11H7ClF3N3O	23576-24-1	2317
Noruron	C13H22N2O	18530-56-8	1966
Novaluron	C17H9ClF8N2O4	116714-46-6	1395
Nuarimol	C17H12ClFN2O	63284-71-9	2390
o,p'-DDD (Mitotane)	C14H10Cl4	53-19-0	2207
o,p'-DDE	C14H8Cl4	3424-82-6	2123
o,p'-DDT	C14H9Cl5	789-02-6	2285
o,p'-Methoxychlor	C16H15Cl3O2	30667-99-3	2388
Octachlorostyrene	C8Cl8	29082-74-4	2053
Octhilinone	C11H19NOS	26530-20-1	1846
Ofurace	C14H16ClNO3	58810-48-3	2331
Omethoate	C5H12NO4PS	1113-02-6	1599
Orbencarb	C12H16ClNOS	34622-58-7	1948
Oryzalin	C12H18N4O6S	19044-88-3	2703
Oxabetrinil	C12H12N2O3	74782-23-3	1848
Oxadiargyl	C15H14Cl2N2O3	39807-15-3	2283
Oxadiazon	C15H18Cl2N2O3	19666-30-9	2204
Oxadixyl	C14H18N2O4	77732-09-3	2290
Oxamyl	C7H13N3O3S	23135-22-0	1541
Oxycarboxin	C12H13NO4S	5259-88-1	2418
Oxychlordane	C10H4Cl8O	27304-13-8	2062
Oxyfluorfen	C15H11ClF3NO4	42874-03-3	2217
p,p'-DDD	C14H10Cl4	72-54-8	2279
p,p'-DDE	C14H8Cl4	72-55-9	2190
p,p'-DDT	C14H9Cl5	50-29-3	2359
Paclobutrazol	C15H20ClN3O	76738-62-0	2125
Paraoxon	C10H14NO6P	311-45-5	1927
Paraoxon-methyl	C8H10NO6P	950-35-6	1818
Parathion	C10H14NO5PS	56-38-2	2001
Parathion-methyl	C8H10NO5PS	298-00-0	1899

Name	Formula	CAS No.	Retention Index
PCNB / Pentachloronitrobenzene (Quintozene)	C6Cl5NO2	82-68-8	1780
PCP / Pentachlorophenol	C6HCl5O	87-86-5	1763
Pebulate	C10H21NOS	1114-71-2	1467
Penconazole	C13H15Cl2N3	66246-88-6	2066
Pencycuron	C19H21ClN2O	66063-05-6	1686
Pendimethalin (Penoxalin)	C13H19N3O4	40487-42-1	2065
Pentachloroaniline	C6H2Cl5N	527-20-8	1860
Pentachloroanisole	C7H3Cl5O	1825-21-4	1731
Pentachlorobenzene	C6HCl5	608-93-5	1533
Pentachlorobenzonitrile	C7Cl5N	20925-85-3	1783
Pentachlorothioanisole	C7H3Cl5S	1825-19-0	1958
Pentanochlor	C13H18ClNO	2307-68-8	1961
Pentoxazone	C17H17ClFNO4	110956-75-7	2563
Perthane (Ethylan)	C18H20Cl2	72-56-0	2246
Pethoxamid	C16H22ClNO2	106700-29-2	2098
Phenanthrene	C14H10	85-01-8	1788
Phenkapton	C11H15Cl2O2PS3	2275-14-1	2496
Phenol	C6H6O	108-95-2	995
Phenothiazine	C12H9NS	92-84-2	2055
Phenothrin(I)	C23H26O3	26002-80-2	2537
Phenothrin(II)	C23H26O3	26002-80-2	2550
Phenthoate (Fenthoate)	C12H17O4PS2	2597-03-7	2090
Phorate	C7H17O2PS3	298-02-2	1702
Phorate oxon	C7H17O3PS2	2600-69-3	1625
Phorate oxon sulfone	C7H17O5PS2	2588-06-9	1895
Phorate sulfone	C7H17O4PS3	2588-04-7	1987
Phorate sulfoxide	C7H17O3PS3	2588-03-6	1971
Phosalone	C12H15ClNO4PS2	2310-17-0	2560
Phosfolan (Cyolane) (Cylan)	C7H14NO3PS2	947-02-4	2073
Phosmet (Imidan)	C11H12NO4PS2	732-11-6	2467
Phosmet oxon (Imidoxon) (Oxoimidan)	C11H12NO5PS	3735-33-9	2357
Phosphamidon	C10H19ClNO5P	13171-21-6	1812
Phthalide	C8H6O2	87-41-2	1360
Phthalimide	C8H5NO2	85-41-6	1465
Picloram-methyl	C7H5Cl3N2O2	14143-55-6	1947
Picolinafen	C19H12F4N2O2	137641-05-5	2486
Picoxystrobin	C18H16F3NO4	117428-22-5	2170
Pindone (Pival)	C14H14O3	83-26-1	1806
Piperalin	C16H21Cl2NO2	3478-94-2	2375
Piperonyl butoxide	C19H30O5	51-03-6	2418
Piperophos	C14H28NO3PS2	24151-93-7	2491
Pirimicarb	C11H18N4O2	23103-98-2	1862
Pirimicarb-desmethyl	C10H16N4O2	30614-22-3	1824
Pirimiphos-ethyl (Pirimifos-ethyl)	C13H24N3O3PS	23505-41-1	2049
Pirimiphos-methyl (Pirimifos-methyl)	C11H20N3O3PS	29232-93-7	1963

Name	Formula	CAS No.	Retention Index
Plifenate (Penfenate)	C10H7Cl5O2	21757-82-4	1901
Prallethrin	C19H24O3	23031-36-9	2112
Pretilachlor	C17H26ClNO2	51218-49-6	2191
Probenazole	C10H9NO3S	27605-76-1	1967
Prochloraz	C15H16Cl3N3O2	67747-09-5	2745
Procymidone	C13H11Cl2NO2	32809-16-8	2100
Prodiamine	C13H17F3N4O4	29091-21-2	1965
Profenofos	C11H15BrClO3PS	41198-08-7	2183
Profluralin	C14H16F3N3O4	26399-36-0	1795
Prohydrojasmon	C15H26O3	158474-72-7	1820
Promecarb	C12H17NO2	2631-37-0	1701
Prometon	C10H19N5O	1610-18-0	1750
Prometryn	C10H19N5S	7287-19-6	1926
Propachlor	C11H14ClNO	1918-16-7	1620
Propamocarb	C9H20N2O2	24579-73-5	1398
Propanil	C9H9Cl2NO	709-98-8	1880
Propaphos	C13H21O4PS	7292-16-2	2123
Propaquizafop	C22H22ClN3O5	111479-05-1	3304
Propargite	C19H26O4S	2312-35-8	2406
Propazine	C9H16ClN5	139-40-2	1766
Propetamphos (Tsar)	C10H20NO4PS	31218-83-4	1790
Propiconazole(I)	C15H17Cl2N3O2	60207-90-1	2353
Propiconazole(II)	C15H17Cl2N3O2	60207-90-1	2367
Propisochlor	C15H22ClNO2	86763-47-5	1925
Propoxur(I)	C11H15NO3	114-26-1	1621
Propyl cresol (Thymol)	C10H14O	89-83-8	1294
Propyzamide (Pronamide)	C12H11Cl2NO	23950-58-5	1792
Proquinazid	C14H17IN2O2	189278-12-4	2403
Prosulfocarb	C14H21NOS	52888-80-9	1935
Prothiofos	C11H15Cl2O2PS2	34643-46-4	2177
Prothoate	C9H20NO3PS2	2275-18-5	1894
Pymetrozine	C10H11N5O	123312-89-0	2177
Pyracarbolid	C13H15NO2	24691-76-7	2028
Pyraclofos	C14H18ClN2O3PS	89784-60-1	2663
Pyraclostrobin	C19H18ClN3O4	175013-18-0	2973
Pyraflufen-ethyl	C15H13Cl2F3N2O4	129630-19-9	2380
Pyrasulfotole	C14H13F3N2O4S	365400-11-9	2151
Pyrazophos	C14H20N3O5PS	13457-18-6	2646
Pyrene	C16H10	129-00-0	2119
Pyributicarb	C18H22N2O2S	88678-67-5	2451
Pyridaben	C19H25ClN2OS	96489-71-3	2724
Pyridafenthion	C14H17N2O4PS	119-12-0	2466
Pyridalyl	C18H14Cl4F3NO3	179101-81-6	2888
Pyridate	C19H23ClN2O2S	55512-33-9	2945
Pyrifenox(I)	C14H12Cl2N2O	88283-41-4	2073

Name	Formula	CAS No.	Retention Index
Pyrifenox(II)	C14H12Cl2N2O	88283-41-4	2126
Pyrifluquinazon	C19H15F7N4O2	337458-27-2	2445
Pyriftalid	C15H14N2O4S	135186-78-6	2646
Pyrimethanil	C12H13N3	53112-28-0	1800
Pyrimidifen	C20H28ClN3O2	105779-78-0	2942
Pyriminobac-methyl	C17H19N3O6	147411-69-6	2382
Pyrimitate	C11H20N3O3PS	5221-49-8	1978
Pyriproxyfen (Pyriproxifen)	C20H19NO3	95737-68-1	2575
Pyroquilon	C11H11NO	57369-32-1	1786
Quinalphos (Diethquinalphione)	C12H15N2O3PS	13593-03-8	2089
Quinoclamine (ACN)	C10H6ClNO2	2797-51-5	1962
Quinomethionate (MQD)	C10H6N2OS2	2439-01-2	2104
Quinoxyfen	C15H8Cl2FNO	124495-18-7	2346
Quizalofop-ethyl	C19H17ClN2O4	76578-14-8	2855
Rabenzazole	C12H12N4	40341-04-6	1999
Resmethrin(I)	C22H26O3	10453-86-8	2410
Ronoxon	C8H8Cl3O4P	3983-45-7	1867
Rotenone	C23H22O6	83-79-4	3321
Schradan (OMPA)	C8H24N4O3P2	152-16-9	1746
Sebuthylazine	C9H16ClN5	7286-69-3	1845
Sebuthylazine-desethyl	C7H12ClN5	37019-18-4	1760
Secbumeton	C10H19N5O	26259-45-0	1824
Sethoxydim	C17H29NO3S	74051-80-2	2392
Siafluofen	C25H29FO2Si	105024-66-6	2900
Simazine	C7H12ClN5	122-34-9	1747
Simeconazole(I)	C14H20FN3OSi	149508-90-7	1907
Simeton	C8H15N5O	673-04-1	1721
Simetryn	C8H15N5S	1014-70-6	1907
Spirodiclofen	C21H24Cl2O4	148477-71-8	2707
Spiromesifen	C23H30O4	283594-90-1	2453
Spirotetramat	C21H27NO5	203313-25-1	2790
Spiroxamine(I)	C18H35NO2	118134-30-8	1898
Spiroxamine(II)	C18H35NO2	118134-30-8	1953
ß-BHC	C6H6Cl6	319-85-7	1760
ß-Endosulfan	C9H6Cl6O3S	33213-65-9	2255
Sulfentrazone	C11H10Cl2F2N4O3S	122836-35-5	2508
Sulfotep	C8H20O5P2S2	3689-24-5	1697
Sulprofos	C12H19O2PS3	35400-43-2	2317
Swep (MCC)	C8H7Cl2NO2	1918-18-9	1753
Tau-fluvalinate(I)	C26H22ClF3N2O3	102851-06-9	2998
Tau-fluvalinate(II)	C26H22ClF3N2O3	102851-06-9	3008
TBP / Tributylphosphate	C12H27O4P	126-73-8	1653
TBZ / Thiabendazole	C10H7N3S	148-79-8	2068
TCMTB (Busan (30))	C9H6N2S3	21564-17-0	2151
TCNB / Tecnazene	C6HCl4NO2	117-18-0	1613

Name	Formula	CAS No.	Retention Index
Tebuconazole(I)	C16H22ClN3O	107534-96-3	2389
Tebufenpyrad	C18H24ClN3O	119168-77-3	2509
Tebupirimfos	C13H23N2O3PS	96182-53-5	1851
Tebutam	C15H23NO	35256-85-0	1696
Tebuthiuron	C9H16N4OS	34014-18-1	1523
Tefluthrin	C17H14ClF7O2	79538-32-2	1837
Telodrin (Isobenzan)	C9H4Cl8O	297-78-9	2011
Temephos (Abate)	C16H20O6P2S3	3383-96-8	3309
TEPP / Tetraethyl diphosphate	C8H20O7P2	107-49-3	1579
TERBA / Terbuthylazine	C9H16ClN5	5915-41-3	1786
Terbacil	C9H13ClN2O2	5902-51-2	1825
Terbufos	C9H21O2PS3	13071-79-9	1785
Terbufos sulfone	C9H21O4PS3	56070-16-7	2066
Terbumeton	C10H19N5O	33693-04-8	1770
Terbumeton-desethyl	C8H15N5O	33124-62-8	1667
Terbuthylazine-desethyl	C7H12ClN5	30125-63-4	1687
Terbutol (Terbucarb)	C17H27NO2	1918-11-2	1901
Terbutryn	C10H19N5S	886-50-0	1952
Terthiophene	C12H8S3	1081-34-1	2218
Tetrachlorvinphos (Dietreen T)	C10H9Cl4O4P	22248-79-9	2139
Tetraconazole	C13H11Cl2F4N3O	112281-77-3	2019
Tetradifon	C12H6Cl4O2S	116-29-0	2536
Tetramethrin	C19H25NO4	7696-12-0	2471
Tetrasul	C12H6Cl4S	2227-13-6	2302
Thanite	C13H19NO2S	115-31-1	1846
Thenylchlor	C16H18ClNO2S	96491-05-3	2391
Thiacloprid	C10H9ClN4S	111988-49-9	2923
Thiamethoxam	C8H10ClN5O3S	153719-23-4	2032
Thiazopyr	C16H17F5N2O2S	117718-60-2	1998
Thifluzamide	C13H6Br2F6N2O2S	130000-40-7	2227
Thiobencarb (Benthiocarb)	C12H16ClNOS	28249-77-6	1976
Thiocyclam (Evisekt)	C5H11NS3	31895-21-3	1502
Thiometon	C6H15O2PS3	640-15-3	1721
Thionazine (Zinophos)	C8H13N2O3PS	297-97-2	1615
Tifatol (Cymiazole)	C12H14N2S	61676-87-7	1908
Tiocarbazil(I)	C16H25NOS	36756-79-3	2012
Tiocarbazil(II)	C16H25NOS	36756-79-3	2015
Tolclofos-methyl	C9H11Cl2O3PS	57018-04-9	1908
Tolfenpyrad	C21H22ClN3O2	129558-76-5	3131
Tolylfluanide	C10H13Cl2FN2O2S2	731-27-1	2075
Toxaphene Parlar 26	C10H10Cl8	142534-71-2	2468
TPPA / Triphenyl phosphate	C18H15O4P	115-86-6	2407
Tralkoxydim	C20H27NO3	87820-88-0	2595
Tralomethrin	C22H19Br4NO3	66841-25-6	3078
Transfluthrin	C15H12Cl2F4O2	118712-89-3	1914

Name	Formula	CAS No.	Retention Index
Triadimefon	C14H16ClN3O2	43121-43-3	2005
Triadimenol	C14H18ClN3O2	55219-65-3	2089
Triallate	C10H16Cl3NOS	2303-17-5	1835
Triamiphos	C12H19N6OP	1031-47-6	2292
Triapenthenol	C15H25N3O	76608-88-3	1966
Triazamate	C13H22N4O3S	112143-82-5	2143
Triazophos	C12H16N3O3PS	24017-47-8	2322
Triazoxide	C10H6ClN5O	72459-58-6	2419
Tribufos	C12H27OPS3	78-48-8	2192
Trichlamide	C13H16Cl3NO3	70193-21-4	2131
Trichlorfon (Dylox) (DEP)	C4H8Cl3O4P	52-68-6	1466
Trichloronate	C10H12Cl3O2PS	327-98-0	2023
Triclopyr 2-butoxyethylester	C13H16Cl3NO4	64700-56-7	2225
Triclopyr-methyl	C8H6Cl3NO3	60825-26-5	1707
Triclosan	C12H7Cl3O2	3380-34-5	2112
Tricyclazole	C9H7N3S	41814-78-2	2175
Tridiphane	C10H7Cl5O	58138-08-2	1926
Trietazine	C9H16ClN5	1912-26-1	1788
Trifenmorph	C23H23NO	1420-06-0	2629
Trifloxystrobin	C20H19F3N2O4	141517-21-7	2373
Triflumizole	C15H15ClF3N3O	68694-11-1	2111
Trifluralin	C13H16F3N3O4	1582-09-8	1689
Trinexapac-ethyl	C13H16O5	95266-40-3	1948
Triphenylmethane	C19H16	519-73-3	2016
Tris(3-Chloropropyl)phosphate	C9H18Cl3O4P	1067-98-7	1806
Tris(b-Chloropropyl)phosphate	C9H18Cl3O4P	6145-73-9	1820
Triticonazole(I)	C17H20ClN3O	131983-72-7	2550
Uniconazole-P(I)	C15H18ClN3O	83657-22-1	2191
Valone	C14H14O3	83-28-3	1845
Vamidothion	C8H18NO4PS2	2275-23-2	2131
Vernolate	C10H21NOS	1929-77-7	1453
Vinclozolin	C12H9Cl2NO3	50471-44-8	1900
Warfarin	C19H16O4	81-81-2	2691
XMC / 3,5-Xylyl methylcarbamate	C10H13NO2	2655-14-3	1568
Zoxamide	C14H16Cl3NO2	156052-68-5	2429

Table S5: Detection frequency of the compounds selected for method validation, when spiked at two concentration/mass levels.

Name	Detection frequency (%)							
	PDMS		POCIS		MSPD Method A		MSPD Method B	
	**5 ng**	**50 ng**	**2.5 ng**	**25 ng**	**2 ng/g**	**20 ng/g**	**1 ng/g**	**10 ng/g**
4-Methylphenol	100	100	100	100	100	100	nd	nd
Benzothiazole	100	100	100	100	100	100	100	100
2,6-Di-tert-butyl-1,4-benzoquinone (BHT-Q)	100	100	100	100	nd	nd	100	100
2-Ethylhexyl salicylate	100	100	0	100	100	100	nd	nd
2,6-Di-tert-butyl-4-methylphenol (BHT)	100	100	100	100	0	100	100	100
Diethyl phthalate	100	100	100	100	100	100	100	100
Benzophenone	100	100	100	100	nd	nd	100	100
Tri-n-butyl phosphate (TnBP)	100	100	100	100	100	100	100	100
Benzenesulfonamide	100	100	100	100	100	100	nd	nd
Phenanthrene	100	100	100	100	100	100	100	100
Tris(1-Chloro-2-Propyl) Phosphate (TCPP)	100	100	100	100	100	100	100	100
Galaxolide	100	100	100	100	100	100	100	100
Di-iso-butyl phthalate	100	100	100	100	nd	nd	nd	nd
Di-n-butyl phthalate	100	100	100	100	100	100	nd	nd
Triclosan	100	100	0	0	0	0	100	100
Pyrene	100	100	0	100	100	100	100	100
2-Ethylhexyl 4-methoxycinnamate (EHMC)	100	100	0	0	100	100	100	100
2-Ethylhexyl salicylate, TMS	na	na	60	100	na	na	100	100
Triclosan, TMS	na	na	100	100	na	na	100	100

na: not analysed; nd: not determined due to high concentration in real samples.

Table S6: Compounds found in passive sampler and mussel samples. Coding: 0: not detected; 1: detected; 1S: detected as silylated derivative.

Compound	Type	R1	R2	R3	S1	S2	S3	S4	V1	V2	V3	A1	A2	A3
1,1'-Biphenyl	Industrial chemical	0	0	0	0	0	0	0	0	0	1	0	0	0
1,2,3-Trichlorobenzene	Industrial chemical	0	0	0	0	0	0	0	1	1	1	1	1	1
1,2,4-Trichlorobenzene	Industrial chemical	0	0	0	0	0	0	0	0	0	1	0	0	0
1,6-Diisopropylnapthalene	Pesticides	0	0	0	0	0	0	0	0	0	0	0	1	0
1-Naphthol	Industrial chemical	0	1	0	0	0	0	0	0	0	0	0	0	0
2-(Methylthio)benzothiazole	Pesticide	0	1	0	0	0	0	0	0	0	0	0	0	0
2,2',3,4,4',5'-Hexachlorobiphenyl (PCB 118)	Industrial chemical	0	0	0	0	0	0	0	1	1	1	0	0	0
2,2',4,4',5,5'-Hexachlorobiphenyl (PCB 151)	Industrial chemical	0	0	0	0	0	0	0	1	1	1	0	0	0
2,2',4,4'-Tetrabromodiphenyl ether (BDE 47)	Flame retardant	0	0	0	0	0	0	0	0	0	1	0	0	0
2,2',4,5,5'-Pentachlorobiphenyl (PCB 101)	Industrial chemical	0	0	0	0	0	0	0	1	0	0	0	0	0
2,6-Dibromophenol	Industrial chemical	0	0	0	0	0	0	0	1S	0	0	0	0	0
2,6-Diisopropylnapthalene	Pesticide	0	0	0	1	1	1	1	0	0	0	0	0	0
2,6-Di-tert-butyl-1,4-benzoquinone (BHT-Q)	Transformation product	0	0	0	0	1	0	0	1	0	1	1	1	1
2,6-Di-tert-butyl-4-methylphenol (BHT)	Antioxidant	1	1	1	0	0	0	0	0	0	1	0	0	0
2-Aminobenzothiazol	Industrial chemical	0	0	0	0	0	1S	1S	1S	1S	1S	1S	1S	1S
2-Ethylhexyl 4-methoxycinnamate (EHMC)	UV filter	0	0	0	1	0	1	1	1	1	0	0	0	0
2-Ethylhexyl diphenyl phosphate (EHDPP)	Flame retardant	0	0	0	0	0	0	0	0	1	0	1	1	0
2-Ethylhexyl salicylate	UV filter	0	0	0	0	1	1	1	0	1	0	1	0	0
2-Methylnaphthalene	PAHs	0	0	0	0	0	0	0	1	0	1	0	0	0
2-Methylphenol	Pesticides	0	0	0	0	0	0	0	1	1	1	1	1	0
3,4-DCA / 3,4-Dichloroaniline	Pesticides	0	0	0	0	1	0	0	0	0	0	0	0	0
3,5-Di-tert-butyl-4-hydroxybenzaldehyde (BHT-CHO)	Transformation product	0	0	0	1	0	1	1	0	0	0	0	0	0
4-Chlorophenol	Pesticides	0	0	0	0	0	0	0	1S	1S	0	0	1S	0
4-Hydroxybenzoic acid	Multiple sources	1	1	1	0	0	1	1	0	0	0	0	0	0
4-Methylphenol	Industrial chemical	1	0	0	0	0	0	0	1	1	1	0	1	0
Anthracene	PAH	0	0	0	0	0	0	0	1	1	0	0	1	0

Compound	Type	R1	R2	R3	S1	S2	S3	S4	V1	V2	V3	A1	A2	A3
Benzenesulfonamide	Transformation product	0	0	0	0	1	0	0	1	1	1	1	1	0
Benzophenone	photoinitiator	0	0	0	0	0	1	0	0	0	1	0	0	0
Benzothiazole	Multiple sources	0	0	0	0	1	0	0	1	0	0	0	0	1
Benzyl butyl phthalate	Plastizier	1	0	0	0	0	0	0	0	0	0	0	0	0
Bis(2-ehtylhexyl) adipate	Plasticizer	1	0	0	1	0	0	0	0	1	1	0	0	0
Bis(2-ethylhexyl) phthalate	Plastizier	0	0	0	0	0	0	0	0	1	0	0	0	0
Bisphenol A	Plasticizer	1S	1S	1S	0	1S	1S	1S	0	0	0	0	0	0
Bornyl acetate	Fragance	1	1	1	0	1	0	0	0	0	0	0	0	0
Butylated hydroxyanisole (BHA)	Antioxidant	0	0	0	0	0	0	0	0	1	0	0	0	0
Caffeine	Stimulant	0	1	0	0	0	0	0	0	0	0	0	0	0
Camphor	Fragance	0	0	0	0	0	0	0	0	1	0	0	0	0
DEET / Diethyltoluamide	Pesticide	0	1	0	0	1	0	0	0	0	0	0	0	0
Di-(2-ethylhexyl) terephthalate	Plasticizer	0	0	0	0	0	0	0	0	1	0	1	0	0
Dibutyl phthalate	Plasticizer	1	1	1	1	1	1	1	1	1	1	1	1	0
Dicyclohexyl phthalate	Plasticizer	1	1	1	1	1	1	1	1	1	1	1	1	1
Diethyl phthalate	Plasticizer	1	1	0	1	1	1	1	1	1	1	1	1	0
Diisobutyl phthalate	Plasticizer	1	1	1	1	1	1	1	1	1	1	1	1	0
Dimethyl phthalate	Plasticizer	0	0	0	0	1	0	0	0	0	0	0	0	0
Diphenyl ether	Industrial chemical	0	0	0	0	1	0	0	1	0	1	1	1	1
Galaxolide	Fragance	1	1	1	1	1	0	1	0	0	1	1	1	0
Ibuprofen	Pharmaceutical	0	1S	0	0	1S	0	0	0	0	0	0	0	0
Indole	Natural product	0	0	0	0	0	0	0	1	1	1	1	1	0
Ketoprofen	Pharmaceutical	0	0	0	0	1S	0	0	0	0	0	0	0	0
Metolcarb	Pesticides	0	0	0	0	0	0	0	0	0	0	0	1	0
Musk Ambrette (natural)	Fragance	1	1	0	0	0	0	0	1	1	1	1	1	1
Naphthalene	PAH	0	0	0	0	0	0	0	1	0	0	1	1	0
Naproxen	Pharmaceutical	0	0	0	0	1S	0	0	0	0	0	0	0	0
Octocrylene	UV filter	0	0	0	1	0	1	1	0	0	0	0	0	0

Compound	Type	R1	R2	R3	S1	S2	S3	S4	V1	V2	V3	A1	A2	A3
Oxybenzone (BP-3)	UV filter	0	1	0	0	0	0	0	0	0	0	0	0	0
Paraxanthine	Stimulant	0	0	0	0	0	1S	1S	0	0	0	0	0	0
Phenanthrene	PAH	1	1	1	0	1	0	0	1	0	0	1	1	0
Phenol	Pesticides	0	0	0	0	0	0	0	1	1	1	1	1	0
Phthalide	Industrial chemical	0	0	0	0	0	0	0	0	1	0	0	0	0
Phthalimide	Pesticides	0	0	0	0	0	0	0	1	1	0	1	1	0
Pyrene	PAH	0	0	0	0	1	0	0	1	1	0	1	1	1
Theobromine	Pharmaceutical	0	1S	1S	1S	0	1S	0	0	0	0	0	0	0
Thymol	Fragance	0	0	0	0	0	0	0	0	0	1	0	0	0
Tolytriazole (Methyl-1H-benzotriazole mixture)	Pesticide	0	0	0	0	1	0	0	0	0	0	1	0	0
Tonalide	Fragance	1	1	1	0	1	0	0	0	0	0	0	0	0
Triclosan	Pesticide	0	0	0	0	1S	0	0	0	0	1	1	0	0
Triisobutyl phosphate (TiBP)	Plasticizer	0	1	0	0	1	0	0	0	0	0	0	0	0
Tri-n-butyl phosphate (TnBP)	Plasticizer	0	1	1	0	1	0	0	0	1	0	0	0	0
Triphenyl phosphate	Plasticizer	0	0	0	0	0	0	0	0	1	0	0	0	0
Tris(1-Chloro-2-Propyl) Phosphate (TCPP)	Plasticizer	1	1	1	0	1	0	0	1	1	1	1	1	0
Tris(2-butoxyethyl) phosphate (TBEP)	Plasticizer	0	0	0	0	1	0	0	0	0	0	0	0	0
Venlafaxine	Pharmaceutical	0	1S	0	0	1S	0	0	0	0	0	0	0	0
α-Hexachlorocyclohexane (α-HCH)	Pesticide	0	0	0	0	0	0	0	0	1	1	1	0	0
α-Methylstyrene	Industrial chemical	0	0	0	0	0	0	0	1	1	1	0	1	1
α-Terpineol	Fragance	0	0	0	0	0	0	0	0	0	1	0	0	0

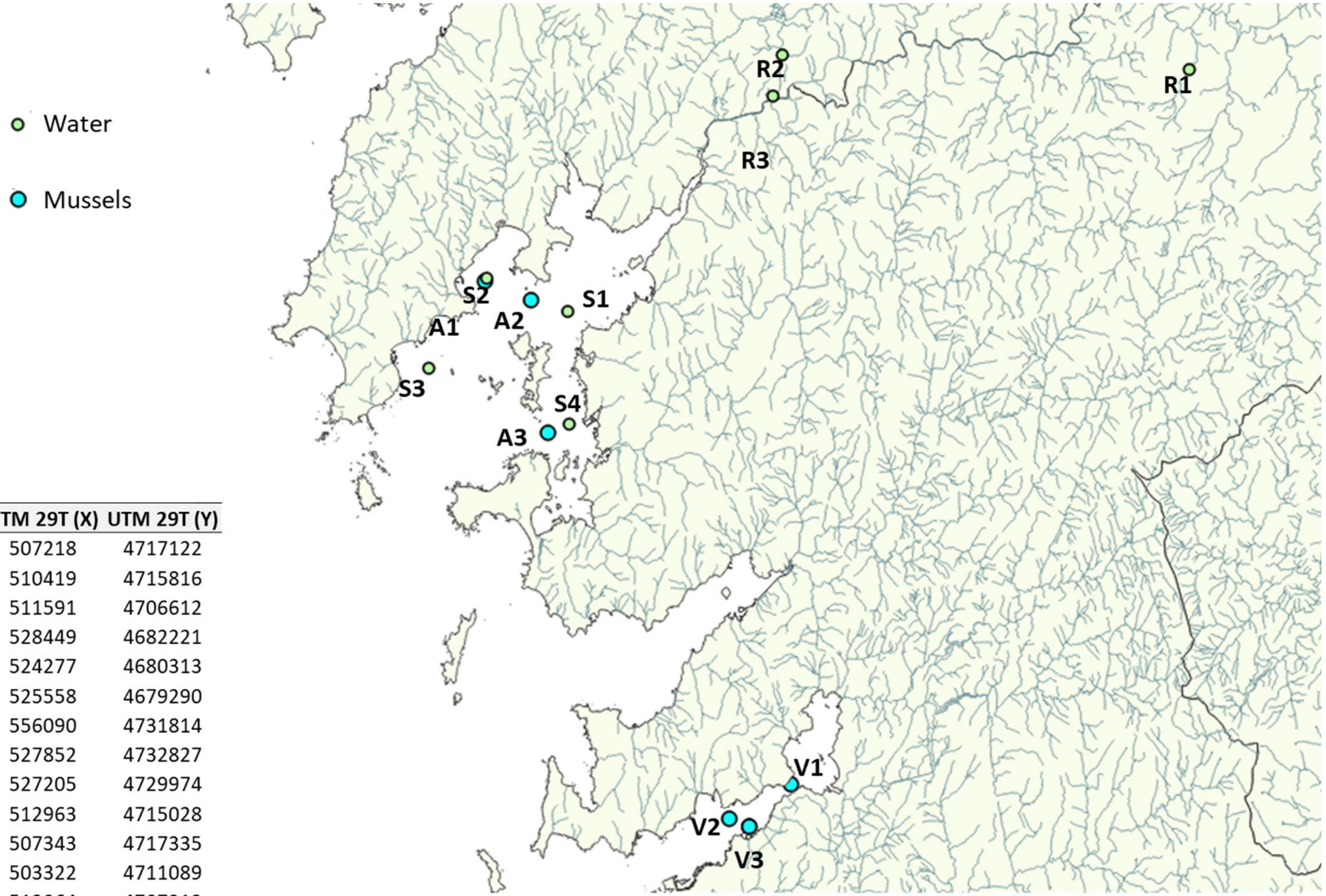

Code	Matrix	Water body	UTM 29T (X)	UTM 29T (Y)
**A1**	Mussel	Ria de Arousa	507218	4717122
**A2**	Mussel	Ria de Arousa	510419	4715816
**A3**	Mussel	Ria de Arousa	511591	4706612
**V1**	Mussel	Ria de Vigo	528449	4682221
**V2**	Mussel	Ria de Vigo	524277	4680313
**V3**	Mussel	Ria de Vigo	525558	4679290
**R1**	River water	Cervantiños creek	556090	4731814
**R2**	River water	Sar river	527852	4732827
**R3**	River water	Sar river	527205	4729974
**S1**	Estuary water	Ria de Arousa	512963	4715028
**S2**	Estuary water	Ria de Arousa	507343	4717335
**S3**	Estuary water	Ria de Arousa	503322	4711089
**S4**	Estuary water	Ria de Arousa	513064	4707213

Figure S1: Location of the samples analyzed in this work

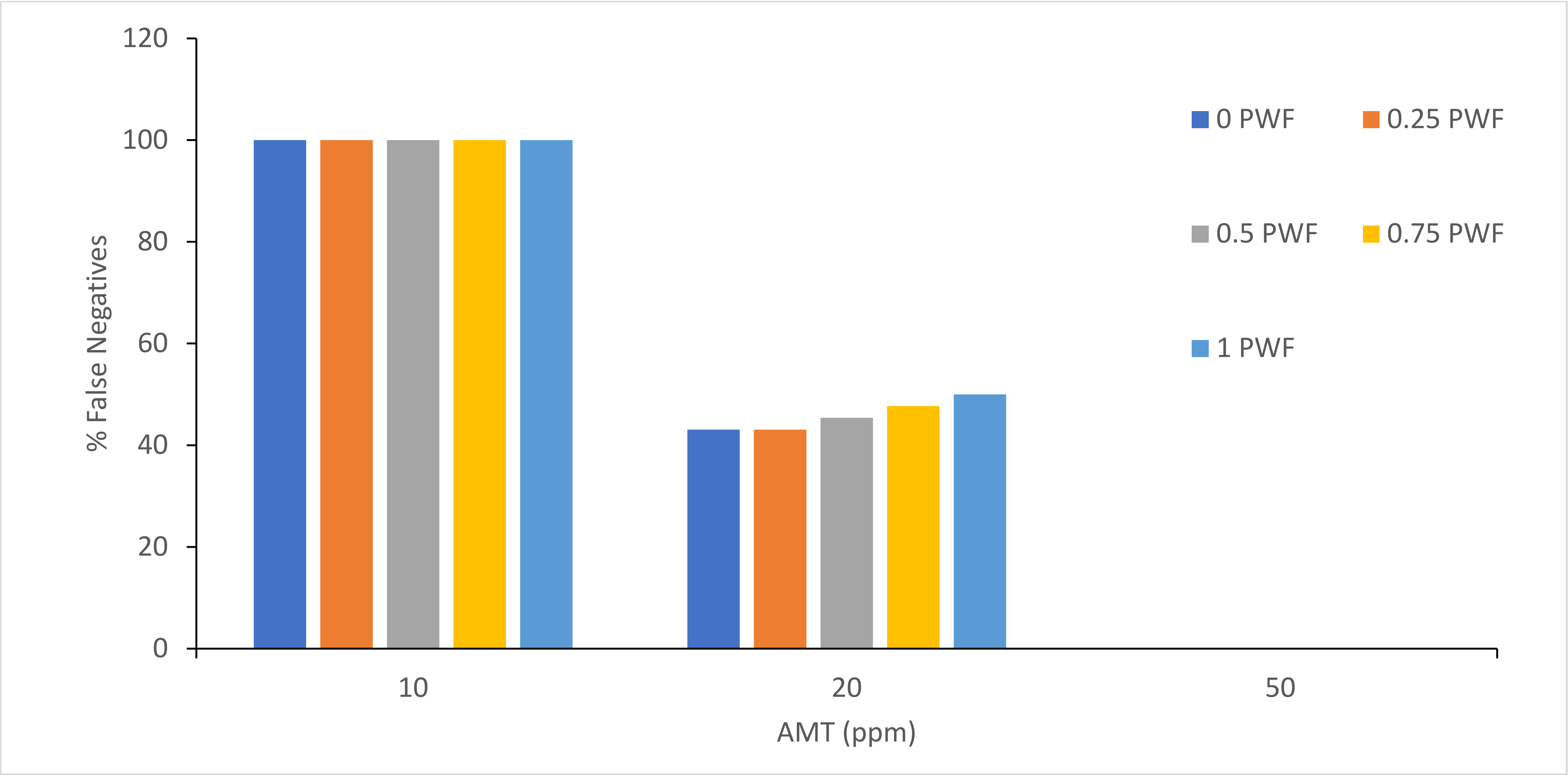

Figure S2: Percentage of false negatives as a function of the accurate-mass tolerance (AMT) for different values of pure weight factor (PWF). Mussel spiked concentration: 100 ng/d dw, equivalent to 500 ng/g in the extract.

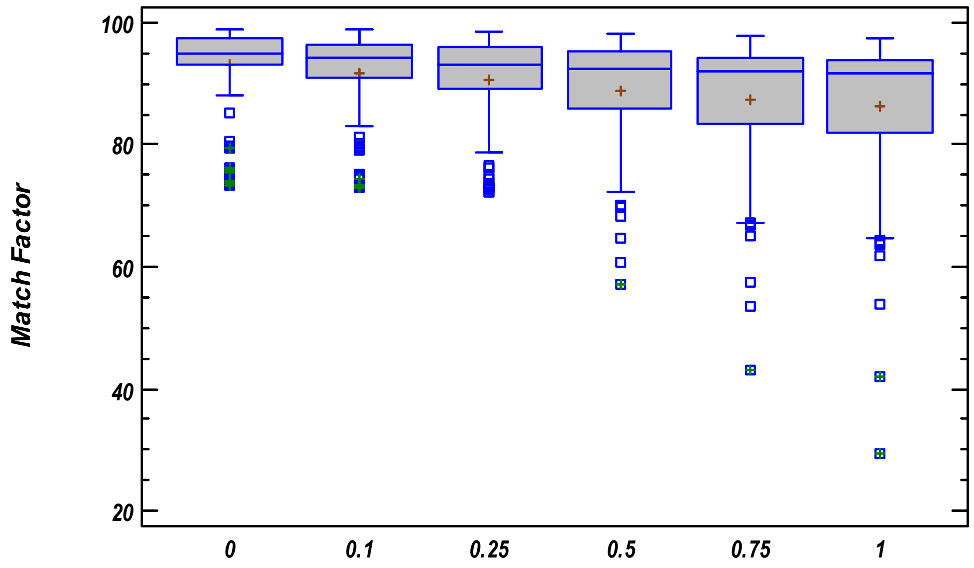

Figure S3: Match factors obtained for the model analytes at different values of pure weight factor (PWF). Mussel spiked concentration: 100 ng/d dw, equivalent to 500 ng/g in the extract.

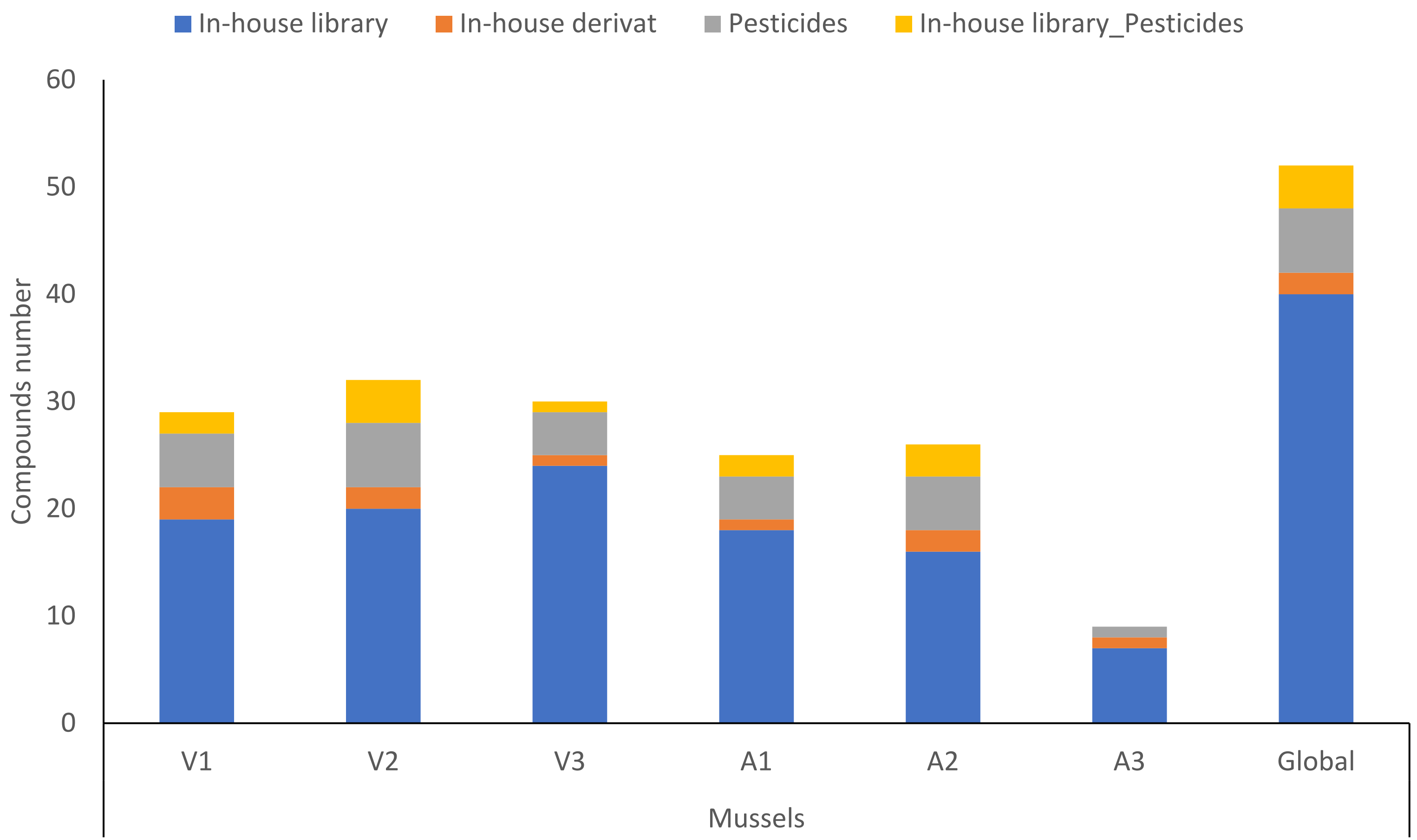

Figure S4: Distribution of compounds detected in the samples according to the library used, (a) for mussel samples

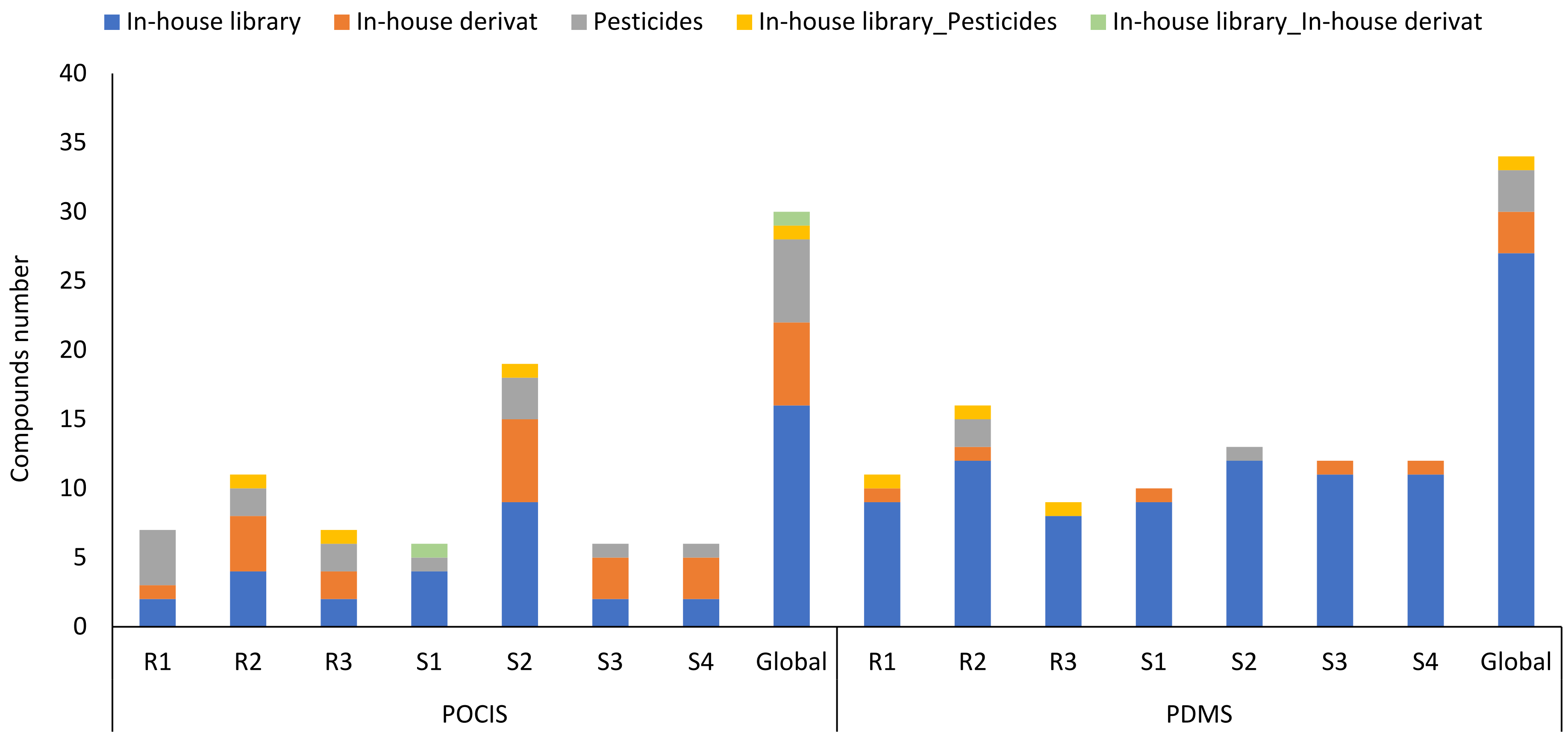

Figure S4: Distribution of compounds detected in the samples according to the library used, (b) in passive samplers

Benzophenone

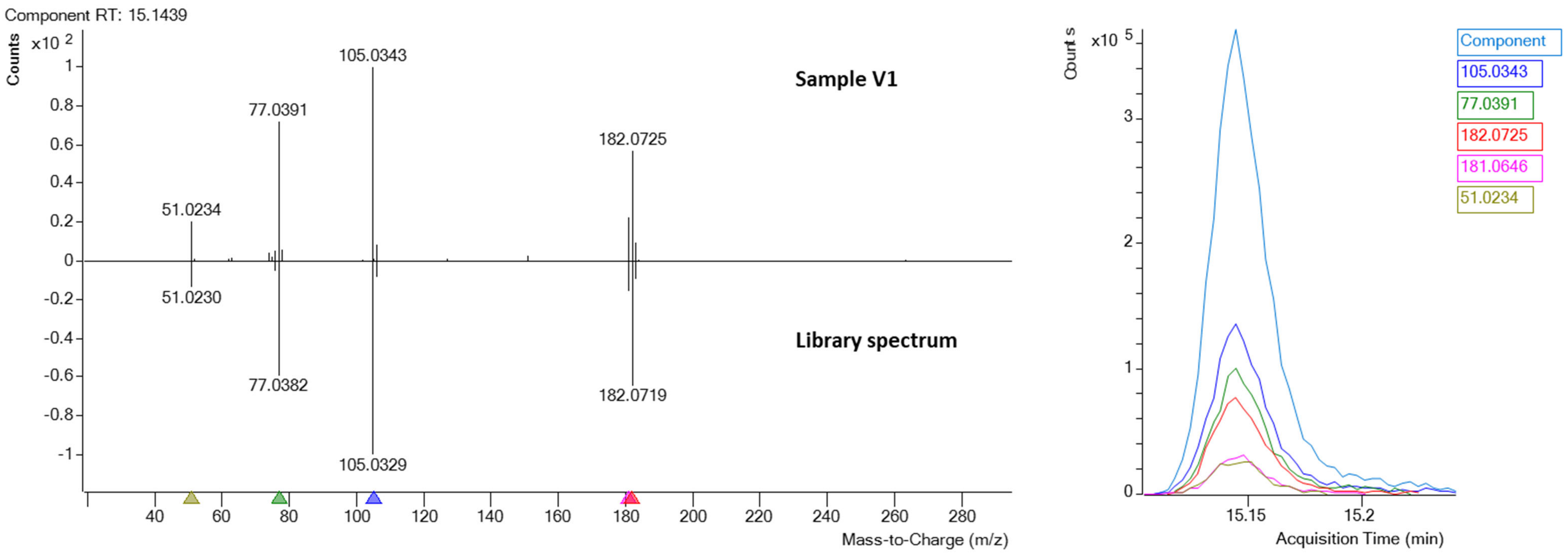

Library RI: 1653 **RI:** 1646 **Delta RI:** 7

Figure S5: Examples of chemicals detected in the samples.

Tri-n-butyl phosphate

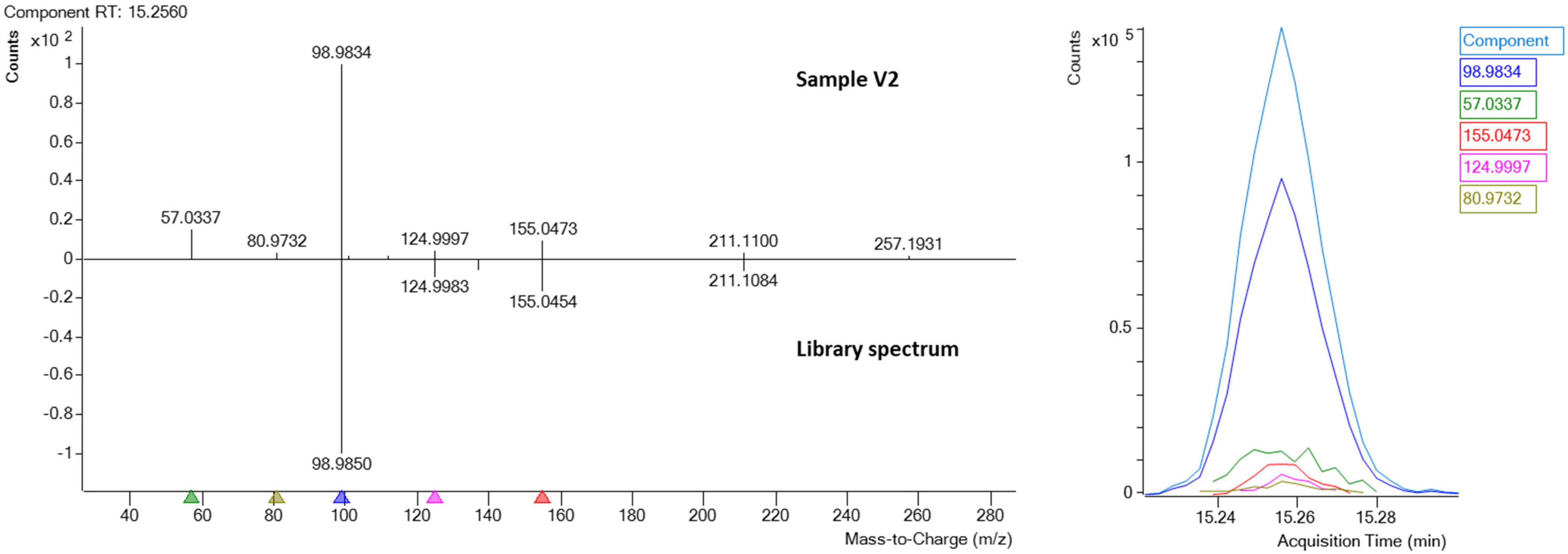

Library RI: 1657 **RI:** 1655 **Delta RI:** 2

Figure S5 (continued): Examples of chemicals detected in the samples.

2-Aminobenzothiazol, TMS derivative

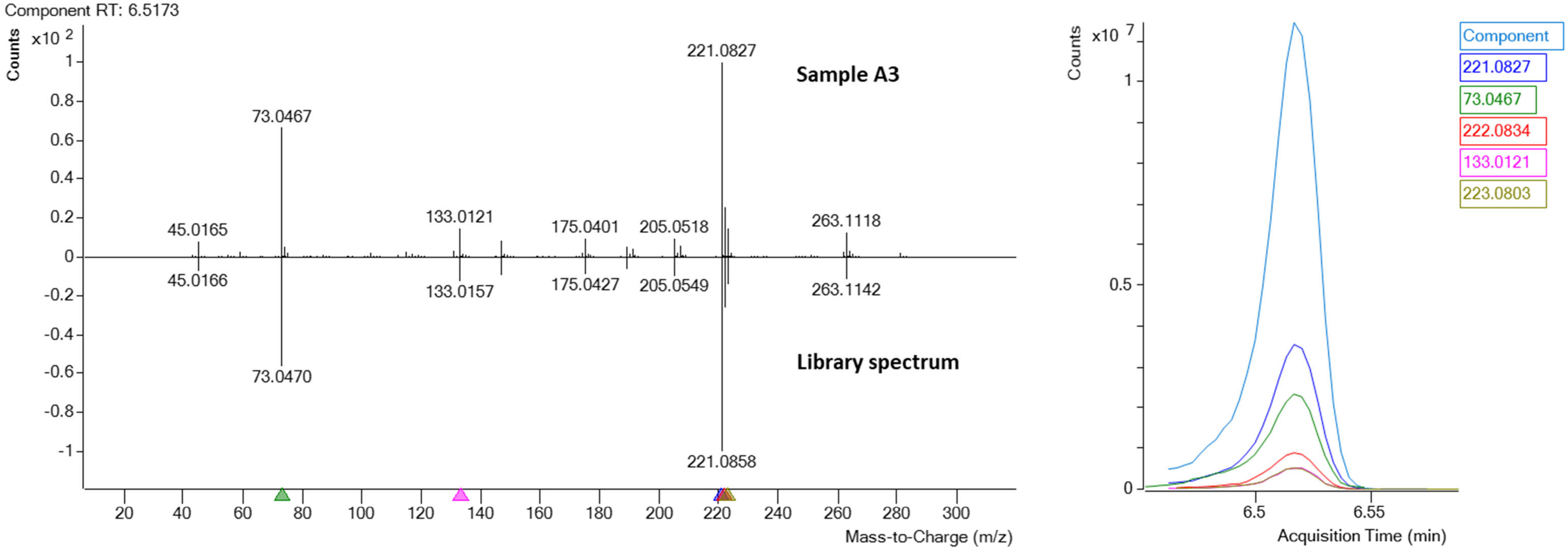

Library RI: 995 **RI:**1005 **Delta RI:** -10

Figure S5 (continued): Examples of chemicals detected in the samples.

BHT-Q

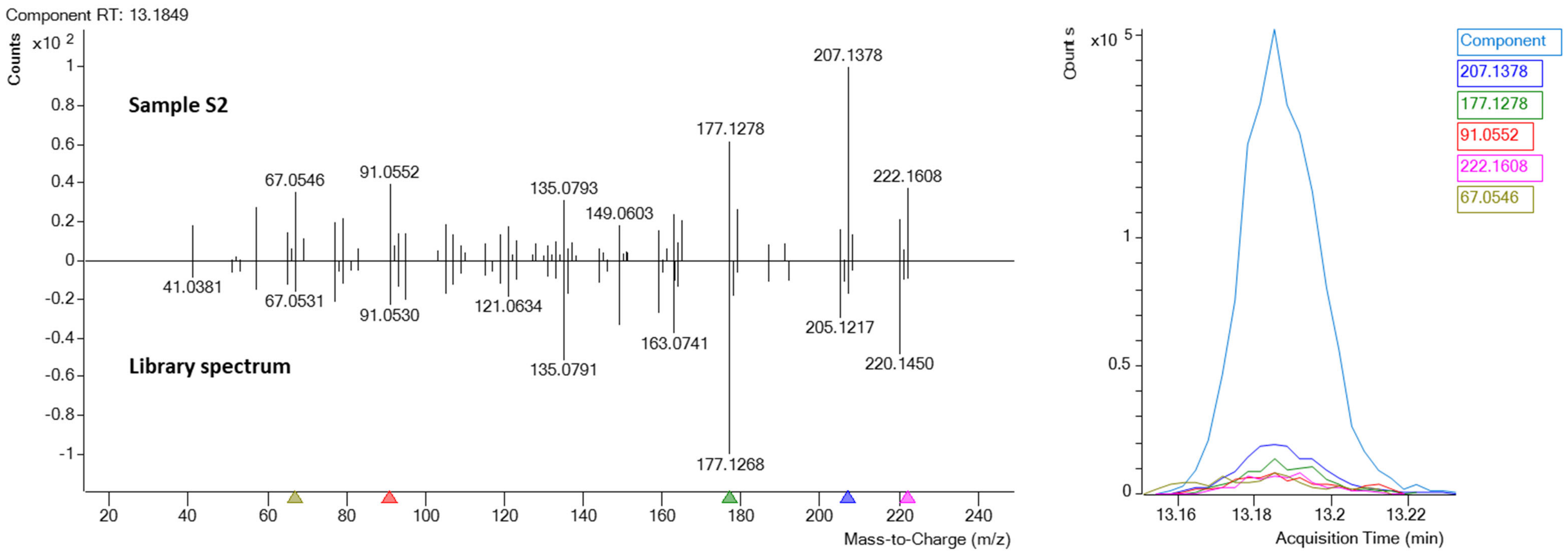

Library RI: 1454 **RI:** 1478 **Delta RI:** -25

Figure S5 (continued): Examples of chemicals detected in the samples.

BHT

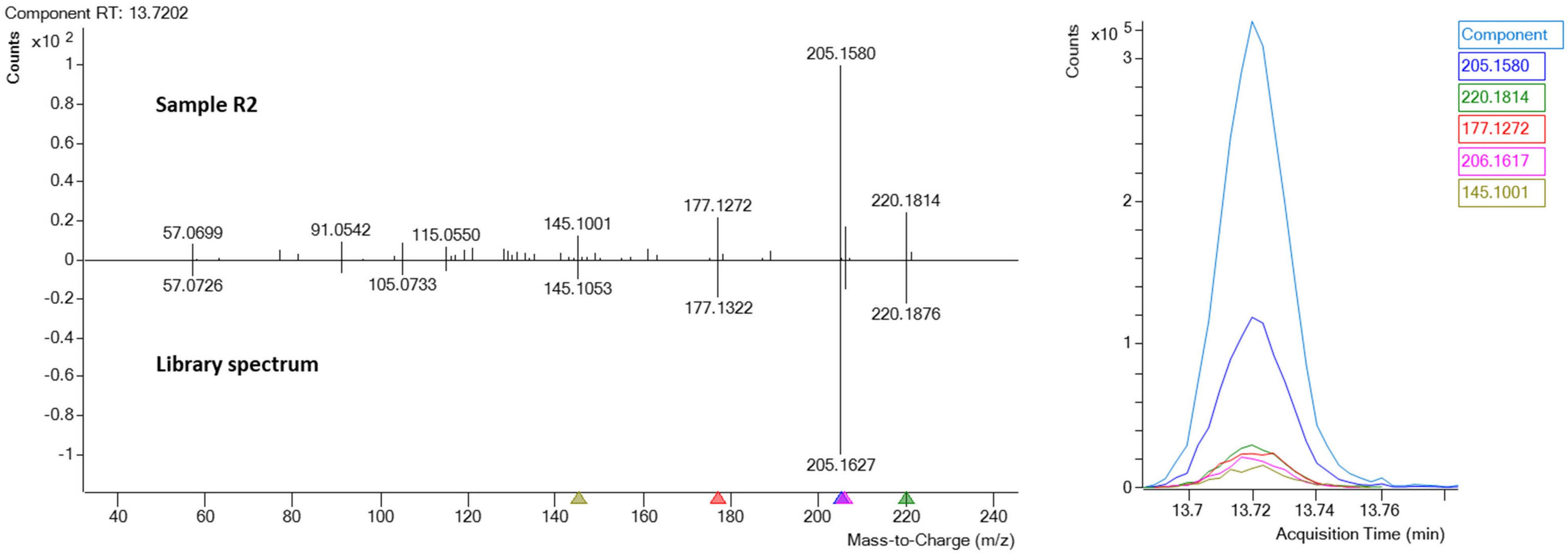

Library RI: 1526 **RI:** 1522 **Delta RI:** 4

Figure S5 (continued): Examples of chemicals detected in the samples.

Bisphenol A, 2TMS derivative

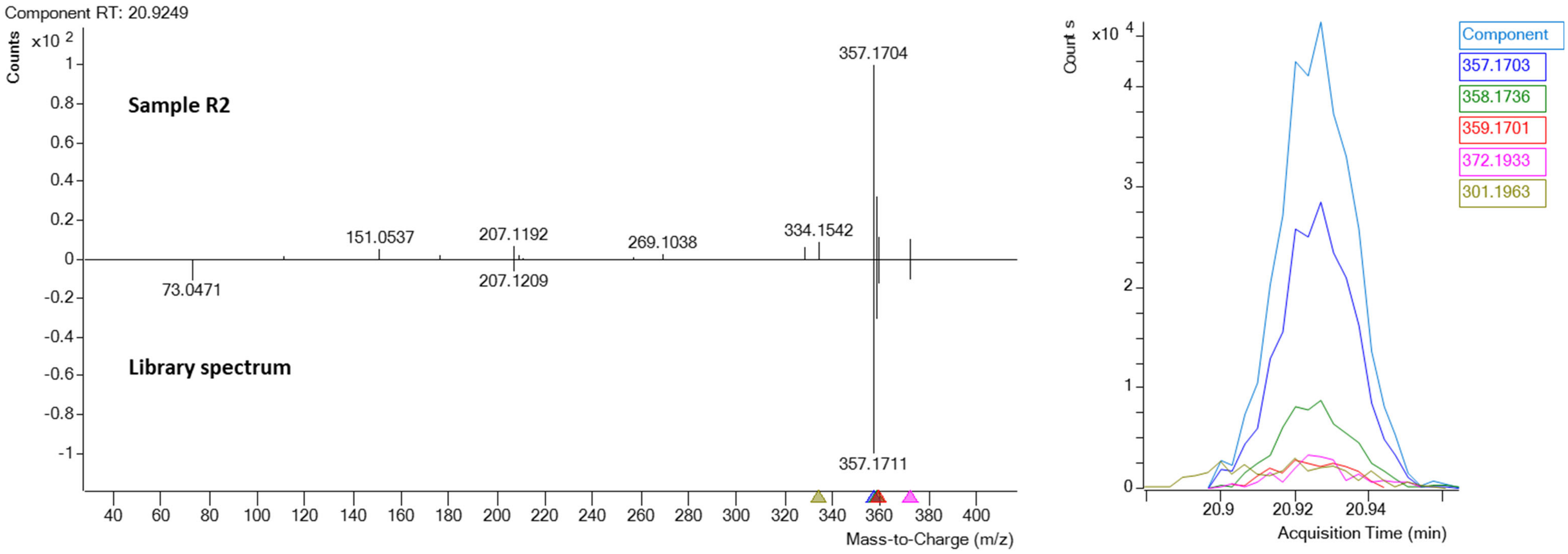

Library RI: 2236 **RI:** 2234 **Delta RI:** 2

Figure S5 (continued): Examples of chemicals detected in the samples.

Galaxolide

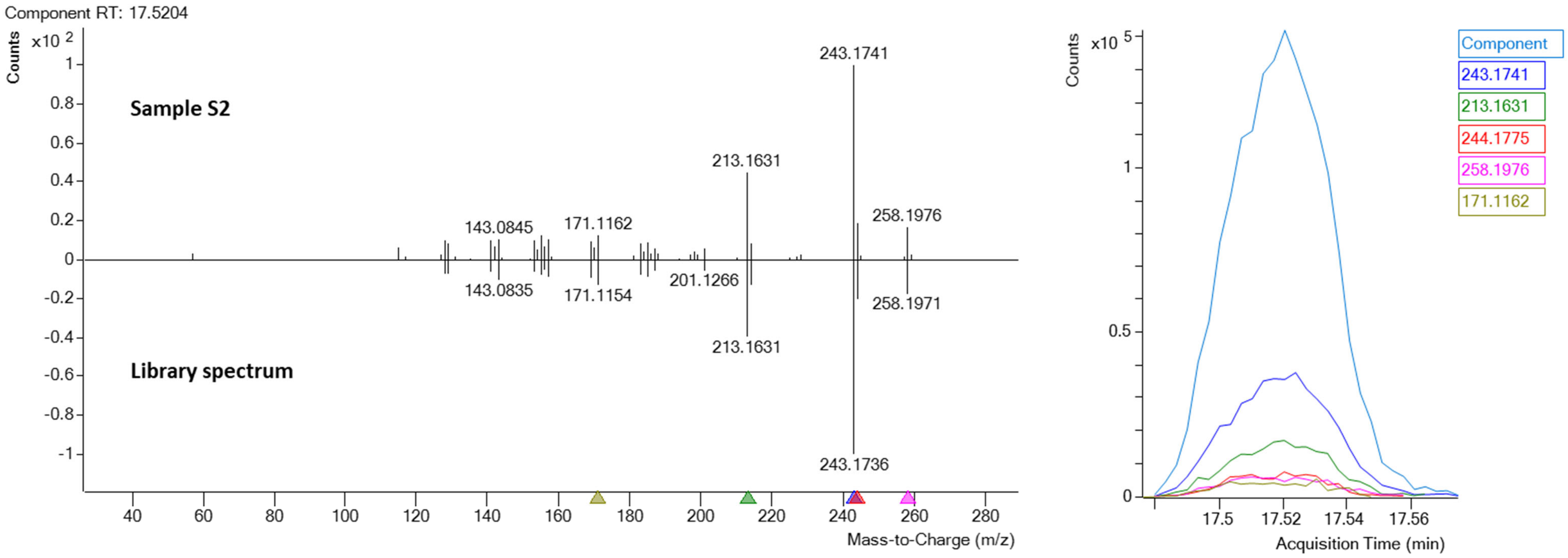

Library RI: 1864 **RI:** 1872 **Delta RI:** -7

Figure S5 (continued): Examples of chemicals detected in the samples.
